# The skull variation of the olive field mouse *Abrothrix olivacea* (Cricetidae: Abrotrichini) is localized and correlated to the ecogeographic features of its geographic distribution

**DOI:** 10.7717/peerj.15200

**Published:** 2023-04-14

**Authors:** Marcial Quiroga-Carmona, Pablo Teta, Guillermo D’Elía

**Affiliations:** 1 Instituto de Ciencias Ambientales y Evolutivas, Facultad de Ciencias, Universidad Austral de Chile, Valdivia, Región de los Ríos, Chile; 2 Colección de Mamíferos, Facultad de Ciencias, Universidad Austral de Chile, Valdivia, Región de los Ríos, Chile; 3 School of Biological Sciences, University of Nebraska—Lincoln, Lincoln, Nebraska, United States; 4 División de Mastozoología, Museo Argentino de Ciencias Naturales “Bernardino Rivadavia”, Buenos Aires, Buenos Aires, Argentina

**Keywords:** Ecomorphology, Vegetation physiognomy, Environmental variables, Lineal morphometrics, Southern south America

## Abstract

The relationship between phenotypic variation and landscape heterogeneity has been extensively studied to understand how the environment influences patterns of morphological variation and differentiation of populations. Several studies had partially addressed intraspecific variation in the sigmodontine rodent *Abrothrix olivacea*, focusing on the characterization of physiological aspects and cranial variation. However, these had been conducted based on geographically restricted populational samples, and in most cases, the aspects characterized were not explicitly contextualized with the environmental configurations in which the populations occurred. Here, the cranial variation of *A*. *olivacea* was characterized by recording twenty cranial measurements in 235 individuals from 64 localities in Argentina and Chile, which widely cover the geographic and environmental distribution of this species. The morphological variation was analyzed and ecogeographically contextualized using multivariate statistical analyses, which also included climatic and ecological variation at the localities where the individuals were sampled. Results indicate that the cranial variation of this species is mostly clustered in localized patterns associated to the types of environments, and that the levels of cranial differentiation are higher among the populations from arid and treeless zones. Additionally, the ecogeographical association of cranial size variation indicate that this species does not follow Bergmann’s rule and that island populations exhibit larger cranial sizes compared to their continental counterparts distributed at the same latitudes. These results suggest that cranial differentiation among the populations of this species is not homogeneous throughout its geographic distribution, and that the patterns of morphological differentiation are also not completely consistent with the patterns of genetic structuring that have been described recently. Finally, the analyses performed to ponder morphological differentiation among populations suggest that the contribution of genetic drift in the formation of these patterns can be ruled out among Patagonian populations, and that the selective effect imposed by the environment could better explain them.

## Introduction

The influence of landscape heterogeneity on the morphological differentiation of a given species or populations is a longstanding topic of interest in evolutionary biology. In general, studies directed to parsing this interaction first characterize both, the variation in shape and size of morphological structures, and then these are statistically correlated to the variability of different climatical (*e.g*., temperature, precipitation, humidity) and habitat physiognomy aspects (*e.g*., land cover, vegetation coverture, primary productivity; Sikes & Kennedy, 1992; Yom-Tov & Yom-Tov, 2004; Wolf, Friggens & Salazar-Bravo, 2009; McNab, 2010; Marchan-Rivadeneira et al., 2012; García-Mendoza et al., 2018; Ariosa-Olea & Mancina, 2018). Few other studies have also considered aspects, such as insularity (see Li et al. (2021) for a recent assessment on this topic), as potential drivers of phenotypic change. Assessment such as these have demonstrated that morphological variation is seldom homogenous across regions with disparate habitats and/or climates; instead, this component of the intraspecific variation is usually structured according to the pattern of variation of one or several of the environmental aspects scrutinized (see Blackburn & Gaston, 1994; Gaston, Chown & Evans, 2008). Moreover, in some cases it has been described the presence of localized morphotypes (referred as ecotypes) closely associated to the different habitat types occupied by the studied species (*e.g*., Grieco & Rizk, 2010; Cordero & Epps, 2012; Alvarado-Serrano, Luna & Knowles, 2013; Souto-Lima & Millien, 2014). Under the idea that shared genetic composition is largely responsible for phenotypic resemblance (see Maestri et al., 2016), the discrepant patterns of phenotypic variation may emerge in isolated populations only due to the action of genetic drift (Mayr, 1947; Gould & Johnston, 1972; Carson & Templeton, 1984; Thorpe, 1987). However, morphological differences and/or discontinuities may also arise by the effect of different selective forces and not only by neutral evolutionary mechanisms (see DeWitt & Scheiner, 2004; Crispo, 2008; Goldstein & Ehrenreich, 2021). Additionally, phenotypic plasticity may play a fundamental role due to its potential to give rise to differences between populations even if they share the same genetic makeup (Merilä & Hendry, 2014).

Ecomorphological studies centered on mammal species have widely pondered cranial variations, both in shape and size, as a proxy for assessing the influences of environmental factors on structuring of phenotypic variation (Carnivora: Bubadué et al., 2016; Schiaffini et al., 2013; Schiaffini, Segura & Prevosti, 2019; Migliorini, Fornel & Kasper, 2020; Artiodactyla: Hendges, Bubadué & Cáceres, 2016; Chiroptera: Marchan-Rivadeneira et al., 2012; Ariosa-Olea & Mancina, 2018; Cingulata: Feijó, Patterson & Cordeiro-Estrela, 2020; Primates: Cardini, Jansson & Elton, 2007; Cardini & Elton, 2008; Cáceres et al., 2014; Didelphimorphia: López-Fuster et al., 2000; Magnus, Machado & Cáceres, 2017; Bubadué et al., 2021). The skull is a key structure that holds most sensory and some food processing organs, whose intraspecific variation frequently mirrors the influence of the clinal or steep environmental gradients (see Pergams & Ashley, 2001; Pergams & Lawler, 2009; Samuels, 2009; Grieco & Rizk, 2010). Rodent cranial variation has been broadly employed as a proxy to study the ecogeographical association and potential drivers of phenotypic variability in landscapes with natural (*e.g*., Bacigalupe, Iriarte-Díaz & Bozinovic, 2002; Monteiro, Duarte & dos Reis, 2003; Cordero & Epps, 2012; Alvarado-Serrano, Luna & Knowles, 2013; Camargo et al., 2019) or human modified (*e.g*., Martínez et al., 2014; Yalkovskaya et al., 2016; Caccavo et al., 2021; also see references in Coda et al., 2021) environmental configurations.

**Figure 1:**
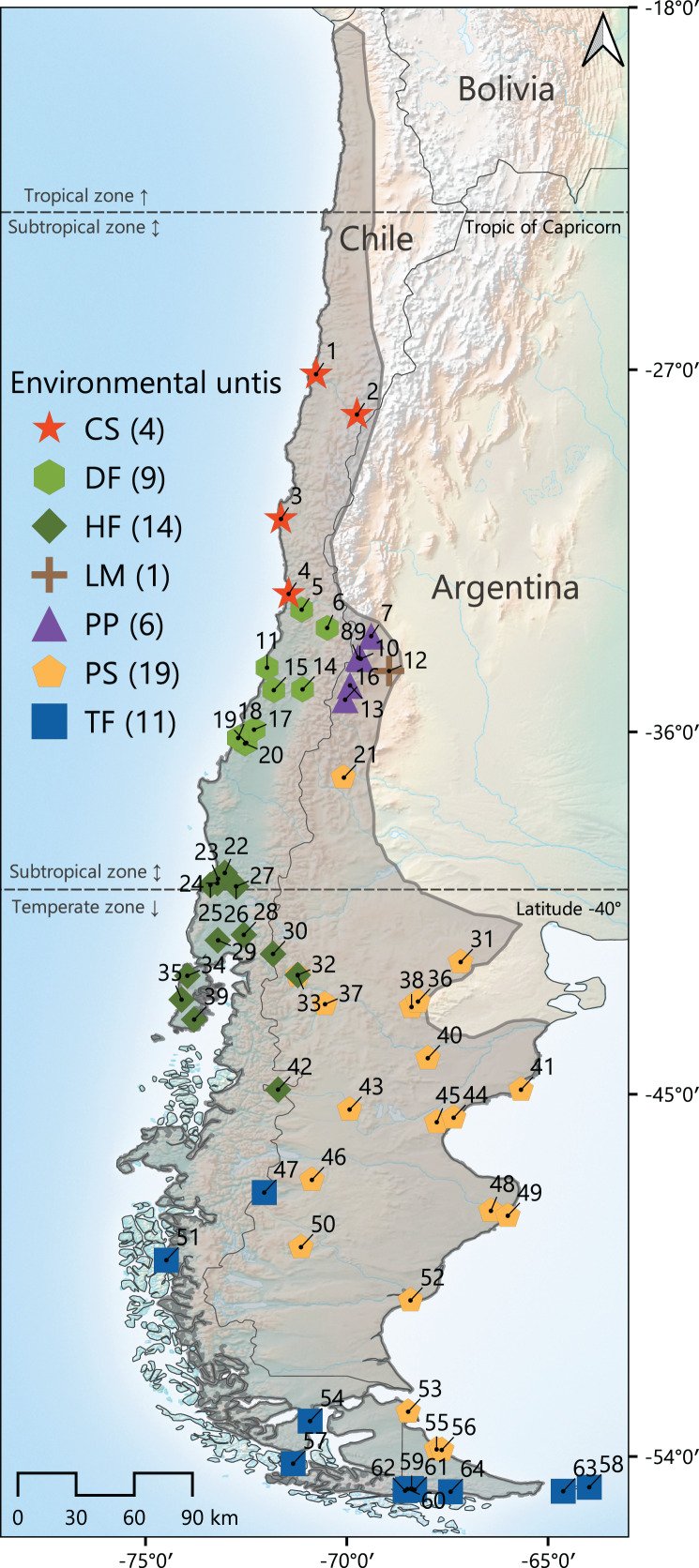
Map of the geographic distribution of *Abrothrix olivacea* throughout southwestern South America. The gray line indicates the approximate distribution of this species. Colored symbols represent the 64 localities where the specimens studied were captured. Localities were latitudinally numbered from north to south. Colors and shapes of these symbols correspond to the environmental units to which localities were assigned. The total number of localities per environmental unit is presented between parenthesis after the corresponding abbreviature, which are as follow: CS, Chilean shrubland; DF, dry forest; HF, humid forest; LM, Low Monte; PP, Andean steppe/Prepuna; PS, Patagonian steppe; TF, temperate forest (see Materials and Methods section).

Geographically widespread species, spanning along several habitat types, ecosystems, or even ecoregions, provide good chances to study how morphological variability is structured throughout heterogeneous landscapes, and in turn, to test whether these patterns of variation correlate with environmental features. The olive field mouse *Abrothrix olivacea* is a suitable model to develop such studies. The geographic distribution of this species occurs throughout a large part of Argentina and almost entirely Chile, extending latitudinally from approximately 19°S (in the Chilean side; 35°S in Argentina) to 55°S and altitudinally from sea level to elevations of about 2,500 m (Fig. 1); the species also distribute in several continental islands of different extensions. Throughout this extensive region the climatic regimes are extremely disparate; for example, the northernmost portion of its distribution occurs along the coastal strip of the Atacama desert, where the average of annual precipitations is about 15 mm, while in southwestern Chile, the distribution lays in lushed forested areas, where the rainfalls can accumulate more than 3,000 mm of rain per year (Moreira-Muñoz, 2011; Veblen, Young & Orme, 2015). As such, *A*. *olivacea* occurs in different types of vegetation physiognomies, including Coastal deserts, Mediterranean shrubs, Valdivian and Magellanic forests, and Andean and Patagonian steppes (Patterson, Teta & Smith, 2015). In turns, across its large geographic range, *Abrothrix olivacea* also overlaps with the several species of sigmodontine rodents, including some species of the genus *Abrothrix* (*e.g*., *A*. *hirta*, *A*. *lanosa*, *A*. *manni*, *A*. *sanborni*; see distributional maps in Teta & Pardiñas, 2014; D’Elía et al., 2015; Patton, Pardiñas & D’Elía, 2015).

The phenotypic variation of *Abrothrix olivacea* has been addressed by several studies, mainly focused on characterizing the anatomical variability of the digestive and excretory systems (*e.g*., Bozinovic et al., 2010, 2011; Naya et al., 2014), as well as the shape and size of skull (*e.g*., Pine, 1973; Yáñez, Valencia & Jaksic, 1979; Patterson, Gallardo & Freas, 1984; Valladares-Gómez et al., 2020; Sánchez et al., 2022). These have demonstrated that variation of the assessed traits is correlated with environmental characteristics, such as water availability and the latitudinal distribution of populations. A possible island effect (see van der Geer, 2018) on the skull morphology of the olive mouse has been suggested by Valladares-Gómez et al. (2020) for a restricted area of Chilean Patagonia. In turn, those assessments centered on cranial and external variation, accounted for a wide variability that was the basis for the recognition of *A*. *olivacea* as a polytypic species (see Mann, 1978; Patterson, Teta & Smith, 2015). However, most of these studies have been based on reduced populational samples, gathered from restricted portions of the species geographic range. Thus, the conclusions that have emerged from these studies can be considered as limited, and larger surveys that globally contextualize the cranial variation with landscape characteristics are still needed. The mentioned aspects were considered to develop this study and linear measurements were taken to characterize the variability on skull shape and size of populations sampled across most of its geographic distribution and covering all environmental settings in which *A. olivacea* is found. Skull variation was correlated with ecological, climatic, and geographic variables (hereafter referred as ecogeographic variables) to explore whether the pattern of variation on this phenotypic component mirrors the environmental heterogeneity and to examine its relationships with ecogeographic dimensions and its correspondence to some ecogeographic rules (*e.g*., Bergmann, Foster). These were discussed with evolutionary, ecogeographical and taxonomical perspectives, considering that to date, the pattern of cranial variation of this species has not been assessed with an extensive geographic sampling under an ecological contextualization.

## Materials and Methods

### Specimens examined and morphological data

Linear skull variation of *Abrothrix olivacea* was characterized examining 235 adult specimens of age classes 3, 4 and 5, according to the pattern of tooth-wear described by Patterson (1992). This material comes from 64 localities (36 in Argentina, and 28 in Chile; see Fig. 1, Supplemental Material, Table S1) and is housed in the following museums and biological collections: Colección de Mamíferos del Instituto Argentino de Investigaciones de las Zonas Áridas, Mendoza, Argentina (CMI); Colección de Mamíferos del Centro Nacional Patagónico, Puerto Madryn, Argentina (CNP); Colección de Mamíferos de la Universidad Austral de Chile, Valdivia, Chile (UACH); Field Museum of Natural History, Chicago, United States (FMNH); Museo de La Plata, La Plata, Argentina (MLP); Museo Argentino de Ciencias Naturales “Bernardino Rivadavia” Ciudad Autonoma de Buenos Aires, Argentina (MACN-Ma); United States National Museum, Washington, D.C., United States (USNM). Twenty craniodental measurements were recorded using a digital caliper to the nearest 0.01 mm, following the definitions provided by Patterson (1992) and Barbero, Teta & Cassini (2020): braincase breadth (BB), condylo-incisive length (CIL), diastema length (DL), frontal length (FL), frontal sinus width (FSW), incisive foramina length (IL), incisive foramina width (IW), interorbital breadth (IB), maxillary toothrow length (TRL), mesopterygoid fossa width (WMF), nasal length (NL), nasal width (NW), palatal width at M1 (PW.M1), palatal width at M3 (PW.M3), palatilar length (PalL), parietal length (ParL), rostrum width (RW), skull length (SL), zygomatic breadth (ZB), zygomatic plate width (ZPW). Prior to the development of the statistical analyses, measurements were log-_10_ transformed to enhance normality and homoscedasticity (see Sokal & Rohlf, 1995; Zar, 1998) and to reduce the negative effects associated to the potential presence of outliers in the main dataset. In addition, a geometric mean transformation was also done over each measurement to reduce potential effects of size differences (see Mosimann & James, 1979).

### Acquisition of ecogeographic data

Geographic coordinates in decimal degrees of sampling localities were taken directly from information provided by collectors. In cases where this information was not available, coordinates were determined with Google Maps, by a georeferencing process in which field notes, collector catalogs and/or museum specimen labels were employed as guide information. Using QGIS 3.18.2-Zürich (Quantum GIS Development Team, 2021), these localities were plotted onto maps that describe the vegetation units present along Argentina and Chile (see Eva et al., 2002, 2004; Luebert & Pliscoff, 2006; Oyarzabal et al., 2018), as well as the life zones and ecoregions occurring in both countries (see Morrone, 2015; Derguy et al., 2019; Derguy, Martinuzzi & Arturi, 2021). Localities were classified and grouped according to the vegetation physiognomy of the habitat present at each site, as the habitats of southern South American small-mammal species are more determined by the growth form of plants (*i.e*., vegetation physiognomy) than by plant species (*i.e*., taxonomic composition) that constitute the botanical community (Monjeau et al., 1997, 1998). Therefore, types of habitats present in sampling localities were generalized into the following vegetation physiognomies/environmental types (hereafter environmental units): Chilean shrubland (CS), which includes desertic and thorny formations; dry forest (DF), which includes sclerophyllous and deciduous forest types; humid forest (HF), such as the Valdivian and others evergreen forests; Low Monte (LM); Andean steppe or Prepuna (PP); Patagonian steppe (PS); and Temperate forest (TF), that corresponds to Magellanic forests (Fig. 1). Then, the R package *raster* (Hijmans et al., 2015) was employed to stack raster layers with a spatial resolution of 30 arc-sec and corresponding to the 19 bioclimatic variables of the WorldClim 1.4 database (Hijmans et al., 2005), and layers containing data on evapotranspiration (Trabucco & Zomer, 2018), primary productivity (Zhang et al., 2017) and elevation, to finally extract the values recorded by each of these parameters at each site. Additionally, the Lang’s index (see Di Castri & Hajek, 1976), corresponding to the ratio of annual precipitation (Bio 12) to annual mean temperature (Bio 1), was estimated as an indirect measure of the humidity present at each site. This ecogeographic dataset was standardized by means of a PCA performed from a correlation matrix (see Legendre & Legendre, 1998).

### Multivariate statistical analyses

Statistical analyses were carried out considering two main approaches. The first aims to evidence cranial differences that potentially exist among populations of *Abrothrix olivacea* occurring at different environmental units, which could imply the detection of particular ecotypes associated to each environmental units considered. This assessment also served to explore potential morphological differences among the populational clusters that can be recognized according to the recent studies about the structure of the genetic variation of this species. On this respect, Giorello, D’Elía & Lessa (2021) based on a panel of single nucleotide nuclear polymorphisms (nuclear SNPs), focused on Patagonian and Fueguian populations, indicates that the genomic variation of *A*. *olivacea* is structured in three main clusters, distributed in: Tongoy, Coquimbo region, Chile (site corresponding to the localities referred to DF), the Valdivian forest (*i.e*., northwest Patagonia; here referred as HF), and continuously throughout mainland Patagonia (except HF) and Tierra del Fuego, along Magellanic forests and Patagonian steppes (here referred as TF and PS, respectively; see Quiroga-Carmona et al., 2022, Fig. 4). Additionally, Quiroga-Carmona et al. (2022, Fig. 4), based on a sampling of the variability exhibited by the cytochrome-b gene (*Cytb*) throughout the entire geographic range of this species, uncovered four main phylogroups, which are mostly allopatric and correspond to: (1) populations from northern Chile (which occur throughout CS and DF; these environmental units partially represent the distribution of the subspecific nominal forms *A*. *o*. *tarapacensis* and *A*. *o*. *olivacea*, respectively), (2) Mendoza province, Argentina (corresponding to populations occurring in LM and PP), (3) central Chile and Argentinean and Chilean Patagonia (represented by populations present in mainland extensions of HF, TF and PS; mostly referable to the nominal forms *A*. *o*. *brachiotis*), and (4) Tierra del Fuego Island and two southernmost mainland localities in the east of the Brunswick Peninsula (represented by populations occurring in TF, which mostly correspond to *A*. *o*. *xanthorhina*). Thus, the grouping of individuals according to the environmental units present in their collection localities, closely considers the schemes derived from Quiroga-Carmona et al. (2022; see also Giorello, D’Elía & Lessa, 2021). Cranial differences or resemblances among these groups were initially explored with a principal component analysis (PCA), performed with a variance-covariance matrix computed based on all craniodental measurements, using the R package *FactoMineR* (Lê, Josse & Husson, 2008). This PCA was employed to visualize how specimens from these groups ordinate in the multivariate space and to minimize the dimensionality of the data by summarizing cranial variation in a reduced number of variables (*e.g*., those principal components whose accumulate variance is over average eigenvalue) used in the successive analyses. Subsequently, the significance of the differences of the established groups was assessed with a permutational multivariate analysis of variance (PERMANOVA) completed with 1 × 10^4^ iterations and a discriminant function analysis (DFA). Both analyses were developed based on PCA scores of the specimens and considering all principal components (*i.e*., 100% of variance accounted) using the R packages *vegan* (Oksanen et al., 2020) and *Mass* (Ripley et al., 2013), respectively. Following this approach, a second DFA was performed to explore potential differences of island samples associated to the nominal forms *hershkovitzi* (Isla Capitán Aracena, locality 57), *llanoi* (Isla de los Estados, localities 58 and 63), and *markhami* (Isla Wellington, locality 51), respect to mainland populations (Fig. 1).

**Figure 4:**
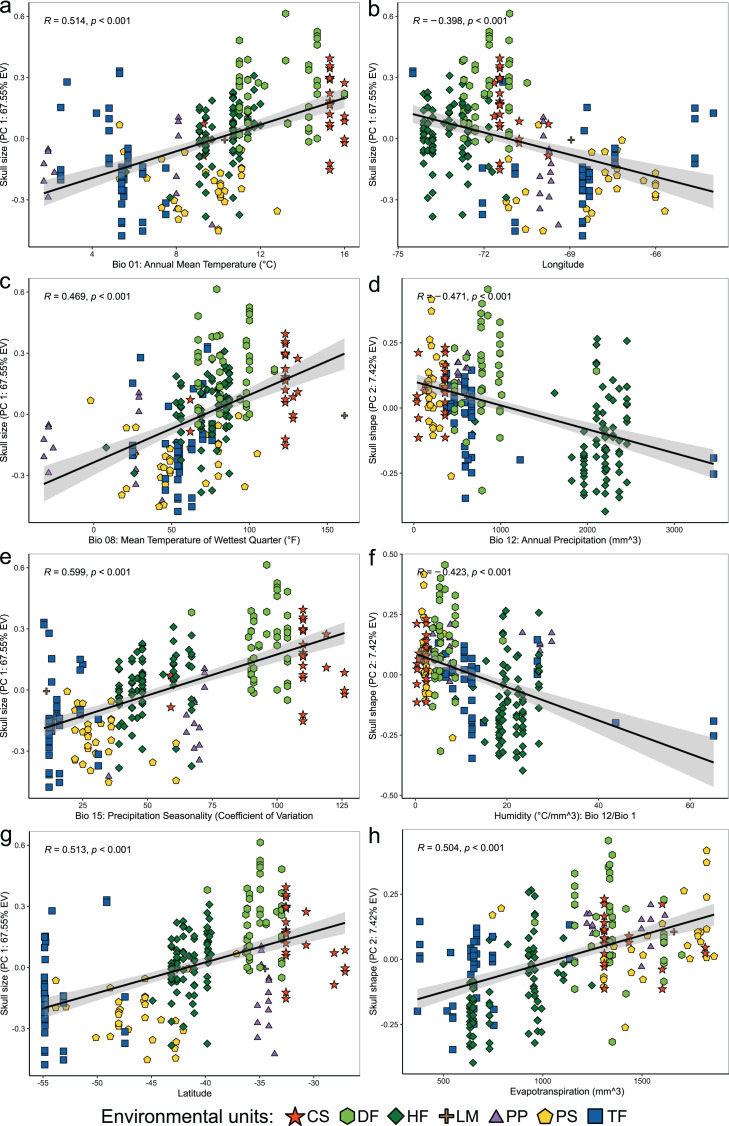
Linear regression analyses showing associations between the scores of cranial variables on PC 1 (summary of cranial size) and PC 2 (summary of cranial shape) alongside to the uncorrelated environmental variables and geospatial variables. Patterns of correlation are only depicted for those variables with significant correlations (*p* < 0.05) and whose patterns of association exhibit remarkable magnitude (*r* values less than −0.40 or greater than 0.40). Correlation patterns of all variables respect to PC1, and PC 2 are present in the Table S3.

The second suite of statistical analyses was focused on exploring associations between cranial measurements and landscape heterogeneity as accounted by the ecogeographic variables analyzed. To avoid redundancy among bioclimatic and ecological variables, levels of paired correlation among these were weighted constructing a correlation matrix based on Pearson’s coefficient, which was subsequently analyzed with the R package *caret* (Kuhn, 2019), to identify and drop those variables with more than 75% of correlation, and then, retain those that represent the dropped ones and constitute the less pair-wise correlated set of variables. Subsequently, associations among the set of uncorrelated variables and cranial measurements were explored performing a canonical correlation analysis (CCA) with the R package *CCA* (González et al., 2008), which also contemplated a geospatial dimension by incorporating elevation, latitude, and longitude in the set of independent variables. The individual interactions between skull size (considered by using PC 1 scores) and shape (considered by using PC 2 scores) and the uncorrelated ecogeographic variables retained were explored performing individual simple linear regressions. These analyses were completed by separately associating the principal component retained (PC 1 or PC 2) with each one of the ecogeographical variables considered employing the following model formula: principal component ~ ecogeographical variable. These explorations were carried out considering the whole set of specimens (*i.e*., at species level) and splitting this dataset according to the environmental units in which populations occur, to uncover the potential patterns that are particularly manifested in each environmental configuration.

Finally, several Mantel tests were performed to evaluate whether the degree of morphological differentiation of individuals is proportional to the geographical distance and/or to the eco-climatic dissimilarities of their localities. Euclidean morphological differences among population localities were calculated using the PCA-standardized scores of specimens in all principal components retained from the previous analysis. Geographic distances among localities were estimated using the latitude and longitude coordinates, while environmental dissimilarities among localities were calculated based on the Gower distance and the uncorrelated set of environmental variables. Mantel tests were performed for the whole dataset and for each environmental unit considered (except in the case of the LM specimens, which come from a single locality; see Fig. 1), by running 1 × 10^4^ randomized permutations and correlating separately the matrix of morphological distance with the matrix of geographic distance and with the matrix of environmental dissimilarity. All these procedures were carried out using *vegan*.

## Results

### Cranial differences among environmental units and island nominal forms

**Figure 2:**
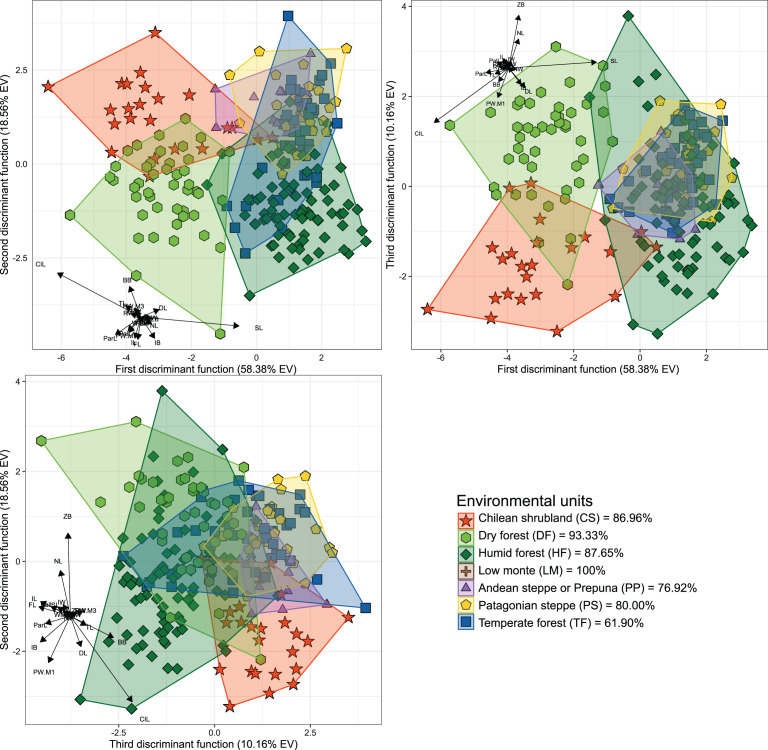
Orthogonal projection of the first three discriminant functions (DF), which explain 87.10% of the among-group variance accounted by the DFA. Individuals were grouped according to the environmental units of their sampling locality (see Fig. 1). Group color-shape codes are explained in the lower right quadrant of the figure. Percentages of correct *a priori* assignation of specimens for each group are also provided in lower right quadrant. Sample sizes of each group are: CS = 23; DF = 45; HF = 81; LM = 1; PP = 13; PS = 30; TF = 42.

The first three dimensions of the PCA account for 79.66% (67.55% for PC 1, 7.42% for PC 2 and 4.69% for PC 3) of the recorded cranial variation. PC1 was highly and positively correlated with most cranial measurements, indicating that scores of this PCA-axis properly summarize the variation associated to skull size. Along this axis, only IB and WMF had correlation values below 0.3 (SM, Table S2), with CIL, ParL, and SL, being the variables with highest loadings (greater than 0.9). PC 2 and PC 3 jointly explain 12.11% of the total variance, and show positive and negative associations with morphological variables, being this more consistent with changes associated with shape rather than size (see Reyment, Blackith & Campbell, 1984). Larger PC 2 values are associated with increases in FL, TRL, RW, ZB, BB and ZPW, and decreases of NL and DL values. In turn, greater values in PC 3 are associated to positive values of FL and IB, and negative values of RW (SM, Fig. S1). Along the plotted PCA axes, the defined groups exhibit high degree of overlap. However, PERMANOVA (Table 1) and DFA (Table 2, Fig. 2) indicated significant statistical differences among most of them. In the first DFA, linear discriminant functions plotted account for 87.10% of the total variance (Table 2), and percentages of correct classification of specimens to each group ranged from 61% to 100% (Fig. 2), with TF obtaining the lowest value and LM the highest. The variables with important contributions (*i.e*., values above 10 or below −10; see F-values in Table 2) to the first discriminant function (DF1) were CIL, ParL, and SL; of these, the two first are negatively associated with this dimension while SL positively associated. In turn, the variables with greater contributions to the second discriminant function (DF2) were CIL and BB; both positively associated with this function. The second DFA performed indicates a better classification (percentages of correct classification ranged from 73.53% to 100%), and those groups from the geographic distributions of the nominal forms *hershkovitzi* (*i.e*., Capitán Aracena Island), *llanoi* (*i.e*., los Estados Island), and *markhami* (*i.e*., Wellington Island) obtained fully correct classifications (SM, Table S3, Fig. S2). In this analysis, the percentage of the variance explained by the first three discriminant functions was 85.24%, and most important variables were the same that of the first DFA, with the exception that in the DF2, IB also is a relevant variable. In general, the linear-cranial configurations present in the environmental units considered (hereafter referred to as ecotypes) are different from each other (*p*-value < 0.05, Table 1), except for the ecotype present in low monte (LM) that only differs from that occurring in Patagonian Steppe (PS). Similarly, no differences were found between the ecotype from temperate forest (TF) with respect to those from LM and Andean Steppe/Prepuna (PP). On this regard, it should be noted that reciprocal differences among ecotypes either present in xeric environments (*i.e*., CS, PP and PS) and/or with more open vegetation (DF) are greater than those among ecotypes from forested areas (*i.e*., HF and TF).

**Table 1:** Results of pairwise permutational multivariate analysis of variance (PERMANOVA) performed to assess the distinction of the groups established based on the distribution of individuals of *Abrothrix olivacea* in the environmental units present throughout the species geographic distribution.

PERMANOVA						
Pairwise comparisons	CS	DF	HF	LM	PP	PS
DF	0.006					
HF	0.001	0.001				
LM	0.544	0.390	0.472			
PP	0.001	0.001	0.002	0.358		
PS	0.001	0.001	0.001	0.033	0.012	
TF	0.001	0.001	0.001	0.371	0.224	0.003

**Note:**

Significant differences are those with *p*-value less than 0.05. Degree of freedom and F-value of this analysis are six and 19.71, respectively. Abbreviation of the environmental units are as follow: CS, Chilean shrubland; DF, dry forest; HF, humid forest; LM, Low Monte; PP, Andean steppe or Prepuna; PS, Patagonian steppe; TF, temperate forest.

**Table 2:** Results of the discriminant function analysis (DFA) performed to explore cranial differences among populations of *Abrothrix olivacea* distributed in the environmental units considered.

Cranial measurements	Linear discriminant functions			F value	*p* value
	DF1	DF2	DF3		
BB: braincase breadth	−4.588	12.976	−3.756	14.504	0.000
CIL: condylo-incisive length	−33.483	21.868	−22.832	24.947	0.000
DL: diastema length	7.401	5.798	−6.79	10.693	0.000
FL: frontal length	−1.137	−9.278	1.129	15.636	0.000
FSW: frontal sinus width	0.534	−1.651	2.653	12.483	0.000
IB: interorbital breadth	5.645	−7.599	−9.163	14.906	0.000
IL: incisive foramina length	−2.351	−9.399	2.311	24.015	0.000
IW: incisive foramina width	0.944	−2.732	2.624	9.218	0.000
NL: nasal length	4.421	−5.969	10.74	6.414	0.000
NW: nasal width	3.642	0.333	0.993	5.169	0.000
PalL: palatilar length	−5.108	−5.516	2.342	25.346	0.000
ParL: parietal length	−10.164	−7.284	−3.435	26.183	0.000
PW.M1: palatal width at M1	−4.754	−2.604	−11.051	34.252	0.000
PW.M3: palatal width at M3	−2.474	2.661	−0.058	18.439	0.000
RW: rostrum width	−4.048	1.289	0.665	24.701	0.000
SL: skull length	39.321	−1.961	4.426	11.975	0.000
TRL: maxillary toothrow length	−6.163	5.292	−1.921	35.243	0.000
WMF: mesopterygoid fossa width	−0.357	−2.065	−0.39	11.002	0.000
ZB: zygomatic breadth	4.485	−6.981	19.961	30.815	0.000
ZPW: zygomatic plate width	−2.274	1.431	1.844	26.376	0.000
Percentage of explained variance (EV)	58.38	18.56	10.16		
Eigenvalues	11.470	6.468	4.784		

**Note:**

Loadings of cranial variables to the first three discriminant functions (DF), F values, and significance (*p* value) obtained for each variable are shown. Percentages of variance explained by each DF and their eigenvalues are provided at the end of the table.

### Associations between cranial variation and environmental and geospatial variables

**Figure 3:**
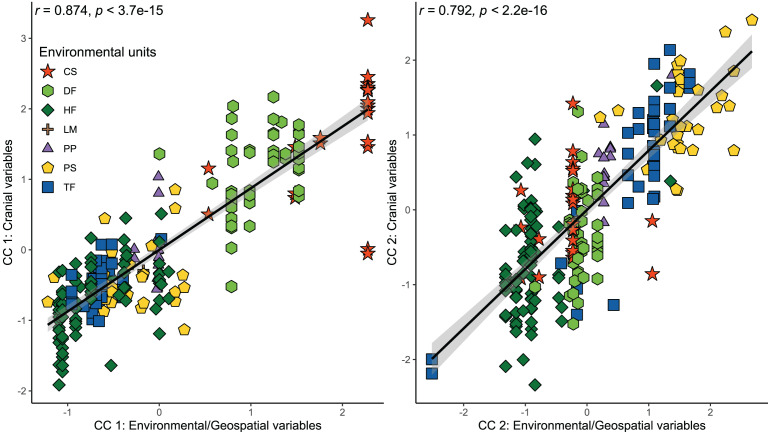
Orthogonal projections of the two first cranial axes against the two first axes that comprise environmental and geospatial variables to represent the canonical correlation among these, addressed throughout a CCA. Coefficients of canonical correlation between axes are shown in Table 3. Colors and shapes of the symbols used to depict specimens are according to the environmental units present in their collecting localities (see Fig. 1).

The results of the CCA indicated a strong and significant association between cranial variables and environmental and geospatial variables (Table 3). The two first canonical functions (CF) obtained high and significant coefficients of canonical correlation of 0.874 and 0.792 (*p* < 0.001), respectively (Fig. 3). The scores of correlations in the first CF indicated that most associations between cranial variables and environmental/geospatial variables are determined by positive relations, being precipitation seasonality (Bio 15), isothermality (Bio 3), latitude, and evapotranspiration, the variables with highest positive loadings (Table 3). In turn, mostly negative associations are indicated by negative scores over second CF, being precipitation of the wettest quarter (Bio 16), annual precipitation (Bio 12), primary productivity, and humidity, the parameters with highest negative loadings. Overall, these correlation patterns are consistent with each other, since the two first CF indicate increases in the values of cranial variables as a function of increases in environmental and geospatial variables. Furthermore, the distribution of specimens constituting each ecotype along the morphological-environmental-geospatial space also shows that the cranial configurations are mostly segregated among environments, with those from treeless areas more segregated from each other than those from woody areas (Fig. 3).

**Table 3:** Results of the canonical correlation analysis (CCA) performed to assess the association between cranial measurements of specimens of *Abrothrix olivacea* and uncorrelated environmental and geospatial variables considered.

Included variables	CF 1	CF 2
*Environmental and geospatial variables*		
Bio 01: Annual Mean Temperature	0.658	−0.367
Bio 03: Isothermality (BIO2/BIO7) (×100)	0.787	0.022
Bio 08: Mean Temperature of Wettest Quarter	0.498	−0.429
Bio 12: Annual Precipitation	−0.476	−0.785
Bio 15: Precipitation Seasonality (Coefficient of variation)	0.863	−0.343
Bio 16: Precipitation of Wettest Quarter	−0.307	−0.821
Humidity	−0.536	−0.582
Evapotranspiration	0.632	0.364
Primary productivity	−0.004	−0.651
Elevation	0.091	0.203
Latitude	0.701	−0.379
Longitude	−0.010	0.733
*Cranial variables*		
BB: braincase breadth	0.571	−0.293
CIL: condylo-incisive length	0.578	−0.583
DL: diastema length	0.135	−0.688
FL: frontal length	0.373	−0.365
FSW: frontal sinus width	0.348	−0.342
IB: interorbital breadth	−0.209	−0.676
IL: incisive foramina length	0.348	−0.650
IW: incisive foramina width	0.053	−0.585
NL: nasal length	0.060	−0.288
NW: nasal width	0.032	−0.269
PalL: palatilar length	0.329	−0.568
ParL: parietal length	0.507	−0.684
PW.M1: palatal width at M1	0.704	−0.434
PW.M3: palatal width at M3	0.514	−0.391
RW: rostrum width	0.681	−0.041
SL: skull length	0.275	−0.685
TRL: maxillary toothrow length	0.798	−0.218
WMF: mesopterygoid fossa width	−0.169	−0.607
ZB: zygomatic breadth	0.731	−0.269
ZPW: zygomatic plate width	0.708	−0.222
Coefficient of canonical correlations	0.874	0.792

**Note:**

The scores of variables in the two first canonical functions and coefficients of correlation between them are provided.

**Figure 5:**
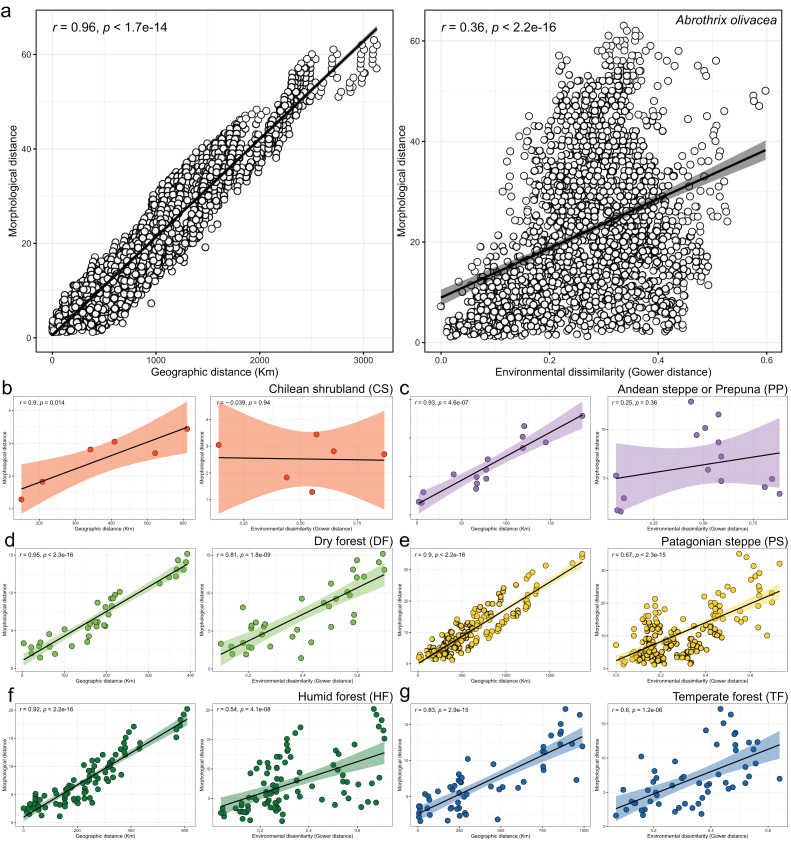
Patterns of association explored with Mantel tests between the morphological and geographic distances (left box) and morphological and environmental dissimilarities (right box). Associations are depicted both, for the analyses performed at the species level (A), and for each environmental units considered (B–G). In the latter plots, dots are colored according to scheme showed in the Fig. 1 and axes equal those of insert a. Spearman correlation coefficients (*R*) and associated *p*-values are shown for each analysis.

Linear regressions also indicated significant trends of ecogeographical associations between cranial variation and some environmental and geospatial variables. Of the 12 variables evaluated, nine are significantly associated (*p* < 0.05) with PC 1, and 11 with PC 2 (Material S4); while only eight of them have remarkable patterns of correlation (*r* values less than −0.40 or greater than 0.40, see Table 4). In general, all these patterns are defined by a positive association, with the exceptions of the negative association of PC 1 with longitude, as well as PC 2 with annual precipitation (Bio 12) and humidity (Fig. 4, Table 4). Regarding PC 1, these positive associations indicate increases in the linear variables defining cranial size as a function of increases in temperature and its seasonality during wet periods, and the seasonality of precipitations. In the geographic context, these cranial variables also exhibit increases associated to lower latitudes, but these decrease toward the east. Respect to PC 2, the linear variables defining cranial shape decrease as a function of rainfall and humidity but show increases in environments with higher primary productivity. It is relevant to note that the significance of these associations is less supported within each of the environmental units, and that in turn, they exhibit more disparate patterns of association (SM, Table S3, Figs. S3 and S4). An example of this can be observed along the annual mean temperature and latitude values. Finally, results of Mantel tests indicated that at the species level, morphological differences among populations increase almost linearly as a function of geographic distance (*r* = 0.96), while environmental dissimilarity has no major influence (*r* = 0.36; Fig. 5). Analyses for each of the recognized environmental units (Figs. 5B–5G) showed that, with a small variation (SD of *r* values is ± 0.041), the almost linear pattern of cranial differentiation in function of the geographic distancing is fulfilled in all populations of the vegetation physiognomies considered. In turn, these analyses also showed that the associations between morphological differences and environmental dissimilarities are less strong and more variable than those mentioned above, and that this relationship is not exhibited by populations from the Chilean shrubland and the Andean steppe or Prepuna (Figs. 5B and 5C).

**Table 4:** Results of linear regressions performed based on scores of the cranial measurements of specimens of *Abrothrix olivacea* in PC 1 and PC 2 and the uncorrelated environmental variables.

Environmental and geospatial variables	PC 1				PC 2			
	Coefficient	SE	*t* value	*p* value	Coefficient	SE	*t* value	*p* value
Bio 01: Annual Mean Temperature	0.245	0.033	7.376	0.000	0.083	0.011	7.562	0.000
Bio 03: Isothermality (BIO2/BIO7) (×100)	0.147	0.024	6.081	0.000	0.051	0.008	6.455	0.000
Bio 08: Mean Temperature of Wettest Quarter	0.026	0.004	6.949	0.000	0.005	0.001	3.489	0.001
Bio 12: Annual Precipitation	0.000	0.000	2.772	0.006	0.000	0.000	−5.671	0.000
Bio 15: Precipitation Seasonality (Coefficient of variation)	0.033	0.003	10.086	0.000	0.010	0.001	8.474	0.000
Bio 16: Precipitation of Wettest Quarter	0.002	0.000	4.528	0.000	0.000	0.000	−3.569	0.000
Humidity	0.015	0.012	1.206	0.229	−0.029	0.004	−7.998	0.000
Evapotranspiration	0.000	0.000	1.297	0.196	0.001	0.000	9.059	0.000
Primary productivity	0.001	0.000	5.928	0.000	0.000	0.000	−1.096	0.274
Elevation	0.000	0.000	−0.864	0.388	0.000	0.000	0.732	0.465
Latitude	0.121	0.015	7.998	0.000	0.036	0.005	7.037	0.000
Longitude	−0.257	0.050	−5.123	0.000	0.004	0.018	0.209	0.835

**Note:**

Regression coefficients (r), standard errors (SE), *t*-value and their significance (*p* value) is provided for the exploration performed at species level. Coefficients of regression and their significance for the exploration performed per environmental units are showed in the Material S3.

## Discussion

The cranial variation of *Abrothrix olivacea* exhibits environmentally localized patterns of differentiation and size changes correlated to several ecogeographical dimensions of the landscape. Ordination analyses suggest that cranial differences among populations occurring in xeric habitats and open vegetation are greater than those observed among populations from forested and humid habitats (Fig. 2). Additionally, correlation analyses shown that cranial differences among populations increase almost lineally with geographic distancing, and with less strength and greater variability in relation to the environmental dissimilarity, being this association comparatively more dissimilar among the environmental units and apparently absent in the Chilean shrubland and Andean steppe/Prepuna (Fig. 5). These analyses also indicate that skull size tend to increase with temperature, rainfall seasonality, and according to a northwesterly geographic direction (Figs. 3 and 4). These findings show that to some extent, the cranial variability of *A*. *olivacea* mirrors the environmental heterogeneity present throughout its geographic range, which in turn could suggest that the ecogeographical dimensions of the landscape have largely influenced the processes that shapes and models the cranial variation of this species. Certainly, some precautions should be considered during the biological interpretation of these results; for example, the results of the PERMANOVAs are sensitive to the size of the analyzed groups (see Anderson & Walsh, 2013); similarly, the significance of the association shown by linear regressions and Mantel tests could be susceptible to sample sizes, ranges of variation analyzed (Jenkins & Quintana-Ascencio, 2020), and spatial autocorrelation (see Crabot et al., 2019). Notwithstanding, this assessment provides insights that are relevant to several issues, ranging from a better understanding of the environmental factors governing the patterns of distribution of the phenotypic variation (see Thorpe, 1987), to emphasizing that conservation plans must consider the biological variability that can exist among and within populations of a same species (see Ryder, 1986; Fraser & Bernatchez, 2001; Thomassen et al., 2011).

Quantitative evaluations of cranial variability have been central in small mammal taxonomy. Over time, other lines of evidence (*e.g*., molecular analysis, ecological niche modeling) have been incorporated to better test hypotheses of species limits, as well as to understand how these biological entities have arisen (see Schlick-Steiner et al., 2010; Carstens et al., 2013). However, few studies with this aim and centered on South American rodent species have extended their taxonomic objectives to explicitly address the associations of the morphological variation characterized with the ecogeographic context in which it occurs (*e.g*., Cadenillas & D’Elía, 2021). Even those that have effectively been aimed at documenting how phenotype varies across the South American landscape are still scarce and necessary, as taxonomic assessment should, ideally, also consider the ecological context in which phenotypic and genetic variation exists (*e.g*., Leaché et al., 2009).

### Environmentally localized variability and cranial differentiation

The phenotypic variation of species with wide geographic distributions and spreading across environmentally heterogeneous landscapes could be deployed in a generalist phenotype or structured into several discrete and environmentally localized phenotypes (see Alvarado-Serrano, Luna & Knowles, 2013). The former is observed in species with wide ecological tolerances, where a single phenotype is functional along all the occupied environmental configurations (Sexton et al., 2017; Des Roches et al., 2018). Conversely, other species tend to show geographically or environmentally discrete patterns of variation, which are localized and associated with specific habitats and/or ecological conditions (*i.e*., ecotypes: Thorpe, 1987; but see Millien et al., 2006). *Abrothrix olivacea* appears to fit better with the second scheme, given that in addition to having reduced home ranges (*ca*. 1,007 m^2^, ±137.9 m^2^, in humid and temperate forests) and low vagility (Meserve, Lang & Murua, 1991; González, Murua & Jofré, 2000), its populations exhibit discrete patterns of cranial variation that are associated to the vegetation physiognomies in which these occurs (see Fig. 2). Although, it should be noted that this pattern cannot be completely generalized to the entire species ecogeographic range, since levels of cranial differentiation were mainly noted among populations occurring in areas with xeric conditions and open vegetation (*i.e*., CS, DF, LM, PP and PS; Fig. 2). Some of the variables (*e.g*., DL, IL, ParL, ZB; see Table S2) that determine these differences are associated with the feeding apparatus, which may suggest that they are largely determined by the particularities of the food resources available at each type of environment/physiognomy (see Samuels, 2009; Cox et al., 2012). The exhibition of these cranial differences is, to some extent, consistent with statements advanced by Monjeau et al. (1997, 1998; see above), since environmental dissimilarities may promote the formation of ecotypes, whereas environments with more similar configurations (*e.g*., Magellanic and Valdivian forests) would not promote ecotypic differentiation (see Alvarado-Serrano, Luna & Knowles, 2013; Souto-Lima & Millien, 2014; Martínez, Sánchez & Sandoval-Salinas, 2022). In addition, this distinction also could suggest that the mechanisms responsible for shaping cranial differences have a stronger influence in populations from open areas (*e.g*., shrub, sclerophyllous, and steppe physiognomies, see Fig. 2).

The assessments of genetic variation of *Abrothrix olivacea* advanced by Giorello, D’Elía & Lessa (2021) and Quiroga-Carmona et al. (2022) have mostly different geographic coverage between each other as well as with that of the present study; however, some of the uncovered cranial discontinuities are consistent with some of the identified genetic breaks. For instance, the genetic studies recognize that populations from northern Chile comprise a distinct genetic group, whose distinction match with cranial differentiation showed by populations that occur in CS and DF (Figs. 1 and 2). In turn, these two populations show a reciprocal degree of cranial differentiation that partially coincides with the genetic differentiation of the mitochondrial lineages referred as *Abrothrix olivacea tarapacensis* and *A*. *o. olivacea* by Quiroga-Carmona et al. (2022; Fig. 4). On the contrary, there is mito-nuclear discordance involving Patagonian and Fueguian populations: Giorello, D’Elía & Lessa (2021) indicate that along this entire region there is two groups disjunctively distributed, one in the Valdivian forests and other continuously distributed along Patagonian steppes and Magellanic forests, while Quiroga-Carmona et al. (2022) describe that a marked genetic discontinuity occurs nearby to the Magellan strait, being a group across open and forested Patagonia and another present in Tierra del Fuego and two southern mainland localities (Quiroga-Carmona et al., 2022: Fig. 4). Irrespective of these genetic inconsistencies, the populations from these regions show no evident cranial differences that match with some of these genetic schemes, as is indicated by the minimal segregation of their polygons along the DFA axes (Fig. 2). Finally, the populations occurring in LM and PP environments, from the area of the mitochondrial lineage restricted to the Argentinian province of Mendoza (see Quiroga-Carmona et al., 2022), only show notable cranial differences with respect to populations from northern Chile, with which is mitochondrially unrelated (no nuclear data is available for these populations).

The mitochondrial patterns of landscape genetic structure described by Quiroga-Carmona & D’Elía (2022) and the results described here consistently indicate that populations of *Abrothrix olivacea* occurring in barren landscapes of northwestern Chile exhibit the highest level of differentiation. Quiroga-Carmona & D’Elía (2022) attributed this fact to the effect imposed by the discontinuous vegetation coverture, arguing that in arid regions mice restrict their activities and movements to the isolated patches of vegetation that provide suitable environmental conditions (see Kelt et al., 1996; Shenbrot, Krasnov & Rogovin, 1999); thus, such environmental circumstances might diminish the already low species vagility (see above). The major effect of the geographic distancing on the cranial differentiation of populations (see Fig. 5), usually invoke the idea of a spatial-neutral process (*e.g*., disruption of gene flow coupled to genetic drift) underlying the observed cranial differences. However, during the development of the correlation analyses, additional Mantel tests were performed following part of the workflow described by Bacigalupe et al. (2008), based on correlate populational means of neutral genetic distances with populational means of phenotypic traits (see Roff & Mousseau, 2005), to address the extent to which genetic drift influence phenotypic differentiation. Given the little coincidence between the genetic and morphological sampling, this analysis was carried out only with populations from Patagonia and demonstrated that there is no correlation between the level of neutral genetic differentiation and its cranial differences (see SM, Fig. S5). This result suggests that—at least among these populations—genetic drift can be ruled out as a main driver of the observed pattern of cranial differentiation (see Roff, 2000; Roff & Mousseau, 2005). As such, it seems more adequate to prioritize selection as responsible for shaping the geographic patterns of cranial variation of *A*. *olivacea*, given that this evolutionary force could explain both, the cranial differences observed among the northern populations, as well as its absence between the genetic clusters present in mainland Patagonia and Tierra del Fuego. Nevertheless, it should be also considered that a potential component of the observed patterns of cranial variation may be due to plasticity, whose load on the phenotype manifestation cannot be directly uncovered with the analyses performed here. To close this part, we state that these our results should be tested with genetic distances gathered from a larger genetic dataset (*e.g*., SNP variation) as ours are based on a single locus. Having said this, we consider that our results represent in a step forward towards understanding the phenotypic evolution of *A. olivacea* and as such, they constitute the basis of the next generation of hypothesis aimed to this goal.

The cranial distinction exhibited by the analyzed island populations have implications that can help to clarify its taxonomic status, as these have been referred to distinct species or as subspecies of *Abrothrix olivacea* (see Patterson, Teta & Smith, 2015; Quiroga-Carmona et al., 2022; Sánchez et al., 2022). The mitochondrial differentiation of populations from the Wellington Island (locality 51; Fig. 1) was shown by Rodriguez-Serrano, Hernández & Palma (2008) and Quiroga-Carmona et al. (2022) and was referred to as *A*. *o*. *markhami*. The second DFA performed (SM, Fig. S2) indicates that this population shows significant levels of cranial distinctiveness (see also Valladares-Gómez et al., 2020); as such, its subspecific status can be maintained. This status should be further evaluated by means of the analysis of nuclear genetic variation. In the case of the populations from the Capitán Aracena and los Estados islands, their cranial distinction was also evidenced by the DFA analysis, and their recognition as subspecific forms of *A*. *olivacea*, *A*. *o*. *hershkovitzi* and *A*. *o*. *llanoi*, respectively, was recently advocated by Sánchez et al. (2022), based on cranial and mitochondrial differences. Quiroga-Carmona et al. (2022) for *hershkovitzi* and Sánchez et al. (2022) for *hershkovitzi* and *llanoi*, consistently describe that although the haplotypes present in both islands were unique and restricted to these places, their genetic differentiation from the Tierra del Fuego populations is low. However, given its geographic isolation and phenotypic distinctiveness, these populations can be considered as evolutionary significant units (see Ryder, 1986; Moritz, 1994), and its recognition as subspecies of *A*. *olivacea* can tentatively be maintained until a more exhaustive revision of their variation allows testing their taxonomic status with a better-sustained approach.

### Ecogeographical associations of cranial variation

The relevance of pondered ecogeographical dimensions (see Table 3) in structuring the cranial variability of *Abrothrix olivacea* was evidenced by the correlational analyses (Figs. 3 and 4). As showed by the trends identified (Table 3; Fig. 4), the magnitudes of the linear cranial dimensions defining their size and shape increase in accordance with increases in temperature, seasonality of precipitations, and evapotranspiration, and decrease according to mean annual precipitation and relative humidity. We note here that by only using PC 2 as a proxy of shape (see Strauss, 2010), not all shape variation among populations, that fraction associated to the remaining PCs, was considering here. Having said that, we found that largest individuals occur in warmer and dryer landscapes, and with sporadic rainfalls (*e.g*., shrublands and dry forests from northern Chile). This trend is also consistent if magnitudes of cranial dimensions are revisited in a northwestern direction along the geographic space (Fig. 4), which is consistent with the geographic pattern of cranial size increase described by Yañez et al. (1978) in populations of *A*. *lanosa* and *A*. *sanborni*, and with the longitudinal west-east pattern of cranial size decrease described by Teta et al. (2022) for *A*. *hirta*.

The identified ecogeographical correlations depict that the changes in cranial dimensions that define the cranial size and shape variation of *Abrothrix olivacea*, conform to or contradict some of the known ecogeographic rules (see Millien et al., 2006 for an exhaustive review of these patterns of phenotypic variation). The identified correlations contradict expected Bergmannian size changes (see Millien et al., 2006), given that according to this rule, species will tend to exhibit larger sizes in higher latitudes and colder climates (see Gaston, Chown & Evans, 2008 and McNab, 2010). An extensive exploration of this pattern conducted by Alhajeri & Steppan (2016; also see Alhajeri, Porto & Maestri, 2020) in over 1,300 rodent species, identified that variables associated with precipitation—especially those determining primary productivity patterns—have the strongest positive association with size changes, describing that in general the Bergman’s rule is not meet by smaller and surface-dwelling rodents, and that food availability rather than heat conservation is the factor with the greatest influence on size variation among species of the order Rodentia. Previously, Yom-Tov & Geffen (2006), focusing on small rodents inhabiting on arid and semi-arid areas described that due to primary productivity is limited by the periodicity of rainfalls, size changes may be strongly determined by food availability, emphasizing that the diet of desert-dwelling rodents consists mainly of annual plants whose abundances are inversely related to rainfalls. Thus, in such habitats increases in rainfalls would imply reductions of food availability for these species. The regions where *A*. *olivacea* has the largest cranial sizes has climatic conditions that are consistent with the climate described by these authors (Fig. 4; SM, Fig. S6), but the association of cranial size with primary productivity does not strictly follow the described trend, as individuals with larger skulls do not occur in the most productive localities (SM, Figs. S3 and S4). In turn, the observed pattern of cranial size increases is not congruent with the positive association described by Yañez et al. (1978) for *A*. *lanosa* and the Chilean populations of *A*. *sanborni*, as well as that described by Teta et al. (2022) for *A*. *hirta*. Nevertheless, interspecific competition, as well as other biotic interactions, can be an additional factor that could come into play for explain this pattern.

Interspecific competition is one of the ecological factors that can modulate the development of body size in terrestrial vertebrates (Lomolino et al., 2012; Benítez-López et al., 2021), and this interaction tends to be more contentious among phylogenetically close related species (see Webb et al., 2002). This ecological phenomenon also can determine that island populations of some species tend to be larger than their mainland counterparts (*i.e*., Foster’s rule, see Millien et al., 2006; Lomolino et al., 2012), given that in areas with low taxonomic diversity, such as islands, biotic communities usually correspond to depauperate assemblages of the nearest continental taxonomic diversity (see Lomolino et al., 2006). Throughout its extensive geographic distribution, *Abrothrix olivacea* coexist with other species of sigmodontine rodents, even with other species of *Abrothrix* (see maps of geographic distribution in Teta & Pardiñas, 2014; D’Elía et al., 2015; Patterson, Teta & Smith, 2015), with which it may compete for the same kind of resources (see Silva, 2005). However, the regions where *A*. *olivacea* exhibits larger cranial sizes also coincide with areas where only one or no other species of *Abrothrix* exist and where the diversity of sigmodontine rodents is low (see Fig. 1; SM, Fig. S6). Thus, it is possible that along these regions where *A*. *olivacea* may be the single species of this genus that constitute the rodent community, it may also reach greater body sizes because competition for resources is lower. This could also explain the larger cranial size observed in the populations from Wellington, Capitan Aracena and Los Estados islands, which reach similar cranial sizes to those observed in the populations from the lowlands of northern Chile (Fig. 1; SM, Fig. S6; see also Valladares-Gómez et al., 2020). Conversely, populations distributed in larger islands such as Chiloé or Tierra del Fuego, exhibit cranial sizes that are equivalent to those shown by continental specimens, possibly because in these larger islands, *A*. *olivacea* cohabit with other sigmodontine rodents, including the congeneric species *A*. *hirta*, *A*. *lanosa*, *A*. *manni*, and/or *A*. *sanborni* (see Patton, Pardiñas & D’Elía, 2015; D’Elía et al., 2015) that in the smaller sized islands does not occur. On this regard, it is necessary to consider that a recent assessment conducted by Alhajeri, Porto & Maestri (2020), indicates that primary productivity, which is a proxy of the supply of resources available to rodent populations, does not play a relevant role driving the geographic variation of body size in rodent species; therefore, the release of competition can be prioritized as one of the explanations to the observed size variations among populations of *A*. *olivacea*. In contrast, Teta et al. (2022) recently shown that skull size of Patagonian populations of *A. hirta* correlates with amount of rain and primary productivity. Therefore, the clear elucidation of these aspects for *A*. *olivacea* will require new studies that should explicitly consider interspecific competence and resource availability as factors influencing the manifestation of the phenotype (see Li et al., 2021).

## Conclusions

The broad geographic coverage of the cranial variation sampling that was employed for this study allowed describing a pattern of phenotypic variation that had not been previously characterized, which indicates that there is not a single trend explaining the pattern of cranial variation of *Abrothrix olivacea* throughout its geographic distribution. In fact, the most evident aspect is that the pattern of cranial variation of this species is not uniform throughout its geographic distribution and that, to some extent, it mirrors the diversity of the South American landscape. The characterization of this wide variability and how it relates to the ecogeographical dimensions of the landscape in which it occurs, provided clues to better understand the phenotypic evolution of this species. It suggests that its extensive cranial variation result from the joint effect of distinct evolutionary and ecological mechanisms. In terms of ecogeographical patterns, these results also evidenced that cranial variation in *A*. *olivacea* is consistent with predictions of the Foster’s rule, but like other species of *Abrothrix*, it does not follow the pattern of size variation predicted by the Bergmann’s rule. However, this intrageneric resemblance does not include the positive association between primary productivity and cranial size, which in *A. olivacea* appears to be better explained by a decrease of interspecific competition. Finally, the uncovered pattern of cranial variation signals the distinction of subspecific forms from northern Chile (*A*. *o. olivacea* and *A*. *o*. *tarapacensis*) and those occurring in insular regions (*A*. *o. hershkovitzi*, *A*. *o*. *llanoi* and *A*. *o*. *markhami*); also evidencing that the discordance between schemes of genetic clustering of the populations from mainland Patagonia and Tierra del Fuego, still require to be studied exhaustively under a genomic approach and a denser geographic sampling that also includes the island populations. Finally, the broad cranial variation of *A*. *olivacea* individualize this sigmodontine species as an interesting model for develop future mechanistic assessments linking ecogeographical dimensions and biotic interactions with the adaptive evolution of morphological aspects.

## Supplemental Information

10.7717/peerj.15200/supp-1 Supplemental Information 1 Raw data: Cranial measurements and climatic data.

10.7717/peerj.15200/supp-2 Supplemental Information 2 Supplementary materials associated with the analyses and results obtained in this work.

## Additional Information and Declarations

### Data Availability

The following information was supplied regarding data availability: The cranial measurements of the individuals studied and the values of the climatic variables characterized in each locality are available in the Supplemental File.
