# Supplementary material for: The skull variation of the olive field mouse Abrothrix olivacea (Cricetidae: Abrotrichini) is localized and correlated to the ecogeographic features of its geographic distribution
Source: PeerJ. 2023 Apr 14;11:e15200. doi: 10.7717/peerj.15200 (PMC10108858; doi:10.7717/peerj.15200)

**SUPPLEMENTARY MATERIALS**

**THE SKULL VARIATION OF THE OLIVE FIELD MOUSE *ABROTHRIX OLIVACEA* (CRICETIDAE: ABROTRICHINI) IS LOCALIZED AND CORRELATED TO THE ECOGEOGRAPHIC FEATURES OF ITS GEOGRAPHIC DISTRIBUTION**

**Marcial Quiroga-Carmona, Pablo Teta, Guillermo D’Elía**

**Table S1.** List of specimens of *Abrothrix olivacea* morphometrically examined. Acronyms are as follow: ARGENTINA (AR): Buenos Aires, Museo Argentino de Ciencias Naturales “Bernardino Rivadavia” (MACN-Ma); Chubut, Puerto Madryn, Colección de Mamíferos del Centro Nacional Patagónico (CNP); La Plata, Museo de La Plata (MLP); Mendoza, Colección de Mamíferos del Instituto Argentino de Investigaciones de las Zonas Áridas, Mendoza, Argentina (CMI). CHILE (CH): Valdivia, Colección de Mamíferos de la Universidad Austral de Chile (UACH). UNITED STATES: Chicago, Field Museum of Natural History (FMNH); Washington, D.C., United States National Museum (USNM). Specimens are sorted according to locality number as shown in Figure 1. Geographical coordinates are provided for each locality.

| **Collection number** | **Locality** | **Locality number** | **latitude** | **longitude** |
| --- | --- | --- | --- | --- |
| FMNH23007 | CH, Atacama, Copiapó, Caldera | Locality 1 | -27.11173 | -70.76631 |
| FMNH23008 | CH, Atacama, Copiapó, Caldera | Locality 1 | -27.11173 | -70.76631 |
| FMNH23009 | CH, Atacama, Copiapó, Caldera | Locality 1 | -27.11173 | -70.76631 |
| FMNH23005 | CH, Atacama, Ramadilla | Locality 2 | -28.11297 | -69.74992 |
| FMNH23006 | CH, Atacama, Ramadilla | Locality 2 | -28.11297 | -69.74992 |
| UACH1419 | CH, Elqui, Quebrada Las Vacas | Locality 3 | -30.70631 | -71.63828 |
| UACH1421 | CH, Elqui, Quebrada Las Vacas | Locality 3 | -30.70631 | -71.63828 |
| UACH72 | CH, Cachagua, Puchuncavi, Valparaiso | Locality 4 | -32.56851 | -71.44615 |
| UACH73 | CH, Cachagua, Puchuncavi, Valparaiso | Locality 4 | -32.56851 | -71.44615 |
| UACH74 | CH, Cachagua, Puchuncavi, Valparaiso | Locality 4 | -32.56851 | -71.44615 |
| UACH74 | CH, Cachagua, Puchuncavi, Valparaiso | Locality 4 | -32.56851 | -71.44615 |
| UACH75 | CH, Cachagua, Puchuncavi, Valparaiso | Locality 4 | -32.56851 | -71.44615 |
| UACH76 | CH, Cachagua, Puchuncavi, Valparaiso | Locality 4 | -32.56851 | -71.44615 |
| UACH77 | CH, Cachagua, Puchuncavi, Valparaiso | Locality 4 | -32.56851 | -71.44615 |
| UACH77 | CH, Cachagua, Puchuncavi, Valparaiso | Locality 4 | -32.56851 | -71.44615 |
| UACH78 | CH, Cachagua, Puchuncavi, Valparaiso | Locality 4 | -32.56851 | -71.44615 |
| UACH79 | CH, Cachagua, Puchuncavi, Valparaiso | Locality 4 | -32.56851 | -71.44615 |
| UACH80 | CH, Cachagua, Puchuncavi, Valparaiso | Locality 4 | -32.56851 | -71.44615 |
| UACH81 | CH, Cachagua, Puchuncavi, Valparaiso | Locality 4 | -32.56851 | -71.44615 |
| UACH81 | CH, Cachagua, Puchuncavi, Valparaiso | Locality 4 | -32.56851 | -71.44615 |
| UACH82 | CH, Cachagua, Puchuncavi, Valparaiso | Locality 4 | -32.56851 | -71.44615 |
| UACH83 | CH, Cachagua, Puchuncavi, Valparaiso | Locality 4 | -32.56851 | -71.44615 |
| UACH84 | CH, Cachagua, Puchuncavi, Valparaiso | Locality 4 | -32.56851 | -71.44615 |
| UACH5364 | CH, Parque Nacional La Campana | Locality 5 | -32.95957 | -71.12328 |
| UACH5365 | CH, Parque Nacional La Campana | Locality 5 | -32.95957 | -71.12328 |
| UACH5366 | CH, Parque Nacional La Campana | Locality 5 | -32.95957 | -71.12328 |
| UACH5367 | CH, Parque Nacional La Campana | Locality 5 | -32.95957 | -71.12328 |
| UACH5368 | CH, Parque Nacional La Campana | Locality 5 | -32.95957 | -71.12328 |
| UACH5369 | CH, Parque Nacional La Campana | Locality 5 | -32.95957 | -71.12328 |
| UACH5370 | CH, Parque Nacional La Campana | Locality 5 | -32.95957 | -71.12328 |
| UACH5371 | CH, Parque Nacional La Campana | Locality 5 | -32.95957 | -71.12328 |
| UACH5372 | CH, Parque Nacional La Campana | Locality 5 | -32.95957 | -71.12328 |
| UACH5601 | CH, Parque Nacional La Campana | Locality 5 | -32.95957 | -71.12328 |
| UACH3614 | CH, Santiago, Las Condes, Los Dominicos | Locality 6 | -33.41462 | -70.48905 |
| CMI7345 | AR, 8 km O Manzano Histórico, Tunuyán, Mendoza [tipo] | Locality 7 | -33.61758 | -69.39704 |
| CMI2166 | AR, 10,5 km a lo largo de RN 40, Laguna del Diamante | Locality 8 | -34.14906 | -69.72142 |
| CMI2167 | AR, 10,5 km a lo largo de RN 40, Laguna del Diamante | Locality 8 | -34.14906 | -69.72142 |
| CMI2188 | AR, 10,5 km a lo largo de RN 40, Laguna del Diamante | Locality 8 | -34.14906 | -69.72142 |
| CMI4953 | AR, San Carlos, Laguna del Diamante, 10.700 pies | Locality 9 | -34.17562 | -69.67534 |
| CMI4926 | AR, San Carlos, 3 km W refugio militar General Alvarado | Locality 10 | -34.17952 | -69.65479 |
| CMI4940 | AR, San Carlos, 3 km W refugio militar General Alvarado | Locality 10 | -34.17952 | -69.65479 |
| UACH87 | CH, Lagunillas, PiCHmu, O'Higgins | Locality 11 | -34.40085 | -71.98429 |
| UACH88 | CH, Lagunillas, PiCHmu, O'Higgins | Locality 11 | -34.40085 | -71.98429 |
| CMI2153 | AR, 40,2 km a lo largo de la R150, 25 de Mayo | Locality 12 | -34.48331 | -68.95051 |
| MLP8-10-2-13 | AR, Laguna El Sosneado, San Rafael, Mendoza | Locality 13 | -34.84192 | -69.92285 |
| MLP8-10-2-14 | AR, Laguna El Sosneado, San Rafael, Mendoza | Locality 13 | -34.84192 | -69.92285 |
| MLP8-10-2-15 | AR, Laguna El Sosneado, San Rafael, Mendoza | Locality 13 | -34.84192 | -69.92285 |
| UACH3754 | CH, Curico, Romeral, Rio Teno | Locality 14 | -34.94024 | -71.10142 |
| UACH3755 | CH, Curico, Romeral, Rio Teno | Locality 14 | -34.94024 | -71.10142 |
| UACH5033 | CH, Fundo Tonlemu, Hualañe, Curico | Locality 15 | -34.95983 | -71.81588 |
| UACH5034 | CH, Fundo Tonlemu, Hualañe, Curico | Locality 15 | -34.95983 | -71.81588 |
| UACH5375 | CH, Fundo Tonlemu, Hualañe, Curico | Locality 15 | -34.95983 | -71.81588 |
| UACH5376 | CH, Fundo Tonlemu, Hualañe, Curico | Locality 15 | -34.95983 | -71.81588 |
| UACH5380 | CH, Fundo Tonlemu, Hualañe, Curico | Locality 15 | -34.95983 | -71.81588 |
| UACH5383 | CH, Fundo Tonlemu, Hualañe, Curico | Locality 15 | -34.95983 | -71.81588 |
| UACH5385 | CH, Fundo Tonlemu, Hualañe, Curico | Locality 15 | -34.95983 | -71.81588 |
| UACH5387 | CH, Fundo Tonlemu, Hualañe, Curico | Locality 15 | -34.95983 | -71.81588 |
| CMI7049 | AR, 1900 m, 10 km S de Las Leñas, Malargue | Locality 16 | -35.19715 | -70.04472 |
| CMI7051 | AR, 1900 m, 10 km S de Las Leñas, Malargue | Locality 16 | -35.19715 | -70.04472 |
| CMI7060 | AR, 1900 m, 10 km S de Las Leñas, Malargue | Locality 16 | -35.19715 | -70.04472 |
| UACH5001 | CH, Fundo Unihue, Cauquenes | Locality 17 | -35.94099 | -72.30704 |
| UACH5002 | CH, Fundo Unihue, Cauquenes | Locality 17 | -35.94099 | -72.30704 |
| UACH5005 | CH, Fundo Unihue, Cauquenes | Locality 17 | -35.94099 | -72.30704 |
| UACH5007 | CH, Fundo Unihue, Cauquenes | Locality 17 | -35.94099 | -72.30704 |
| UACH5008 | CH, Fundo Unihue, Cauquenes | Locality 17 | -35.94099 | -72.30704 |
| UACH5010 | CH, Fundo Unihue, Cauquenes | Locality 17 | -35.94099 | -72.30704 |
| UACH5012 | CH, Fundo Unihue, Cauquenes | Locality 17 | -35.94099 | -72.30704 |
| UACH5013 | CH, Fundo Unihue, Cauquenes | Locality 17 | -35.94099 | -72.30704 |
| UACH1000 | CH, Coelemu, Burca, Fundo la Madera | Locality 18 | -36.14852 | -72.70017 |
| UACH1001 | CH, Coelemu, Burca, Fundo la Madera | Locality 18 | -36.14852 | -72.70017 |
| UACH1002 | CH, Coelemu, Burca, Fundo la Madera | Locality 18 | -36.14852 | -72.70017 |
| UACH1402 | CH, Coelemu, Burca, Fundo la Madera | Locality 18 | -36.14852 | -72.70017 |
| UACH2690 | CH, Coelemu, Burca, Fundo la Madera | Locality 18 | -36.14852 | -72.70017 |
| UACH2692 | CH, Coelemu, Burca, Fundo la Madera | Locality 18 | -36.14852 | -72.70017 |
| UACH4420 | CH, Coelemu, Burca, Fundo la Madera | Locality 18 | -36.14852 | -72.70017 |
| UACH4423 | CH, Coelemu, Burca, Fundo la Madera | Locality 18 | -36.14852 | -72.70017 |
| UACH998 | CH, Coelemu, Burca, Fundo la Madera | Locality 18 | -36.14852 | -72.70017 |
| UACH999 | CH, Coelemu, Burca, Fundo la Madera | Locality 18 | -36.14852 | -72.70017 |
| UACH3769 | CH, Las Eras, Quirihue, Ñuble | Locality 19 | -36.26691 | -72.50117 |
| UACH2392 | CH, Los Remates, Quirihue | Locality 20 | -36.28512 | -72.53117 |
| UACH2395 | CH, Los Remates, Quirihue | Locality 20 | -36.28512 | -72.53117 |
| CNP813 | AR, Volcán Tromen, Neuquén | Locality 21 | -37.12403 | -70.09004 |
| UACH231 | CH, Valdivia, Fundo Rucapangue | Locality 22 | -39.49975 | -73.03484 |
| UACH233 | CH, Valdivia, Fundo Rucapangue | Locality 22 | -39.49975 | -73.03484 |
| UACH192 | CH, Valdivia, Fundo San Martin | Locality 23 | -39.64541 | -73.19988 |
| UACH195 | CH, Valdivia, Fundo San Martin | Locality 23 | -39.64541 | -73.19988 |
| UACH208 | CH, Valdivia, Fundo San Martin | Locality 23 | -39.64541 | -73.19988 |
| UACH232 | CH, Valdivia, Fundo San Martin | Locality 23 | -39.64541 | -73.19988 |
| UACH1012 | CH, Fundo La Dehesa, Valdivia | Locality 24 | -39.75497 | -73.21885 |
| UACH1018 | CH, Fundo La Dehesa, Valdivia | Locality 24 | -39.75497 | -73.21885 |
| UACH1019 | CH, Fundo La Dehesa, Valdivia | Locality 24 | -39.75497 | -73.21885 |
| UACH1022 | CH, Fundo La Dehesa, Valdivia | Locality 24 | -39.75497 | -73.21885 |
| UACH1023 | CH, Fundo La Dehesa, Valdivia | Locality 24 | -39.75497 | -73.21885 |
| UACH229 | CH, Valdivia, Fundo La Mision | Locality 25 | -39.79865 | -73.39219 |
| UACH1369 | CH, Fundo Santa Rosa | Locality 26 | -39.83167 | -72.75187 |
| UACH223 | CH, Fundo Santa Rosa | Locality 26 | -39.83167 | -72.75187 |
| UACH211 | CH, Esc. Normal Huachocopihue | Locality 27 | -39.83382 | -72.74957 |
| UACH212 | CH, Esc. Normal Huachocopihue | Locality 27 | -39.83382 | -72.74957 |
| UACH213 | CH, Esc. Normal Huachocopihue | Locality 27 | -39.83382 | -72.74957 |
| UACH214 | CH, Esc. Normal Huachocopihue | Locality 27 | -39.83382 | -72.74957 |
| UACH215 | CH, Esc. Normal Huachocopihue | Locality 27 | -39.83382 | -72.74957 |
| UACH215 | CH, Esc. Normal Huachocopihue | Locality 27 | -39.83382 | -72.74957 |
| UACH216 | CH, Esc. Normal Huachocopihue | Locality 27 | -39.83382 | -72.74957 |
| UACH220 | CH, Esc. Normal Huachocopihue | Locality 27 | -39.83382 | -72.74957 |
| UACH221 | CH, Esc. Normal Huachocopihue | Locality 27 | -39.83382 | -72.74957 |
| UACH222 | CH, Esc. Normal Huachocopihue | Locality 27 | -39.83382 | -72.74957 |
| UACH1979 | CH, La Picada, Puerto Octay, Osorno | Locality 28 | -41.03686 | -72.56093 |
| UACH1986 | CH, La Picada, Puerto Octay, Osorno | Locality 28 | -41.03686 | -72.56093 |
| UACH1987 | CH, La Picada, Puerto Octay, Osorno | Locality 28 | -41.03686 | -72.56093 |
| UACH1989 | CH, La Picada, Puerto Octay, Osorno | Locality 28 | -41.03686 | -72.56093 |
| UACH2009 | CH, La Picada, Puerto Octay, Osorno | Locality 28 | -41.03686 | -72.56093 |
| UACH234 | CH, La Picada, Puerto Octay, Osorno | Locality 28 | -41.03686 | -72.56093 |
| UACH236 | CH, La Picada, Puerto Octay, Osorno | Locality 28 | -41.03686 | -72.56093 |
| UACH237 | CH, La Picada, Puerto Octay, Osorno | Locality 28 | -41.03686 | -72.56093 |
| UACH238 | CH, La Picada, Puerto Octay, Osorno | Locality 28 | -41.03686 | -72.56093 |
| UACH239 | CH, La Picada, Puerto Octay, Osorno | Locality 28 | -41.03686 | -72.56093 |
| UACH246 | CH, La Picada, Puerto Octay, Osorno | Locality 28 | -41.03686 | -72.56093 |
| UACH1384 | CH, Llanquihue, Frutillar, Los Lagos | Locality 29 | -41.17251 | -73.20641 |
| CMI_FBM470 | AR, Estación de Aforo, río Manso Superior | Locality 30 | -41.51081 | -71.83415 |
| CMI_FBM505 | AR, Estación de Aforo, río Manso Superior | Locality 30 | -41.51081 | -71.83415 |
| CMI181 | AR, Estación de Aforo, río Manso Superior | Locality 30 | -41.51081 | -71.83415 |
| CMI182 | AR, Estación de Aforo, río Manso Superior | Locality 30 | -41.51081 | -71.83415 |
| CNP863 | AR, Subida de las Nacientes, Río Negro | Locality 31 | -41.71271 | -67.17443 |
| CNP331 | AR, El Maitén, Chubut | Locality 32 | -42.03525 | -71.22076 |
| CNP1268 | AR, Estancia El Maitén, Chubut | Locality 33 | -42.04306 | -71.22139 |
| UACH1235 | CH, Chiloe, Fundo El Venado | Locality 34 | -42.05361 | -73.96306 |
| UACH1237 | CH, Chiloe, Fundo El Venado | Locality 34 | -42.05361 | -73.96306 |
| UACH1238 | CH, Chiloe, Fundo El Venado | Locality 34 | -42.05361 | -73.96306 |
| UACH1241 | CH, Chiloe, Fundo El Venado | Locality 34 | -42.05361 | -73.96306 |
| UACH1242 | CH, Chiloe, Fundo El Venado | Locality 34 | -42.05361 | -73.96306 |
| UACH1246 | CH, Chiloe, Fundo El Venado | Locality 34 | -42.05361 | -73.96306 |
| UACH1247 | CH, Chiloe, Fundo El Venado | Locality 34 | -42.05361 | -73.96306 |
| UACH1256 | CH, Chiloe, Fundo El Venado | Locality 34 | -42.05361 | -73.96306 |
| UACH1257 | CH, Chiloe, Fundo El Venado | Locality 34 | -42.05361 | -73.96306 |
| UACH1263 | CH, Chiloe, Fundo El Venado | Locality 34 | -42.05361 | -73.96306 |
| UACH1198 | CH, Chiloe, Chonchi, Cucao | Locality 35 | -42.64518 | -74.10739 |
| UACH1202 | CH, Chiloe, Chonchi, Cucao | Locality 35 | -42.64518 | -74.10739 |
| UACH1207 | CH, Chiloe, Chonchi, Cucao | Locality 35 | -42.64518 | -74.10739 |
| UACH1208 | CH, Chiloe, Chonchi, Cucao | Locality 35 | -42.64518 | -74.10739 |
| UACH1213 | CH, Chiloe, Chonchi, Cucao | Locality 35 | -42.64518 | -74.10739 |
| UACH1214 | CH, Chiloe, Chonchi, Cucao | Locality 35 | -42.64518 | -74.10739 |
| UACH1215 | CH, Chiloe, Chonchi, Cucao | Locality 35 | -42.64518 | -74.10739 |
| UACH1215 | CH, Chiloe, Chonchi, Cucao | Locality 35 | -42.64518 | -74.10739 |
| UACH1217 | CH, Chiloe, Chonchi, Cucao | Locality 35 | -42.64518 | -74.10739 |
| UACH1219 | CH, Chiloe, Chonchi, Cucao | Locality 35 | -42.64518 | -74.10739 |
| UACH1222 | CH, Chiloe, Chonchi, Cucao | Locality 35 | -42.64518 | -74.10739 |
| UACH1225 | CH, Chiloe, Chonchi, Cucao | Locality 35 | -42.64518 | -74.10739 |
| UACH1227 | CH, Chiloe, Chonchi, Cucao | Locality 35 | -42.64518 | -74.10739 |
| UACH1228 | CH, Chiloe, Chonchi, Cucao | Locality 35 | -42.64518 | -74.10739 |
| CNP4337 | CH, Establecimiento La Maroma | Locality 36 | -42.69436 | -68.23391 |
| CNP4549 | CH, Establecimiento La Maroma | Locality 36 | -42.69436 | -68.23391 |
| CNP1284 | CH, Pasarela Piedra Parada, Chubut | Locality 37 | -42.76013 | -70.54493 |
| CNP409 | CH, Pasarela Piedra Parada, Chubut | Locality 37 | -42.76013 | -70.54493 |
| CNP4544 | CH, Caruhé Niyeu | Locality 38 | -42.82907 | -68.39393 |
| UACH1273 | CH, Chiloe, Puerto Carmen | Locality 39 | -43.14052 | -73.79427 |
| UACH1275 | CH, Chiloe, Puerto Carmen | Locality 39 | -43.14052 | -73.79427 |
| UACH1276 | CH, Chiloe, Puerto Carmen | Locality 39 | -43.14052 | -73.79427 |
| UACH1286 | CH, Chiloe, Puerto Carmen | Locality 39 | -43.14052 | -73.79427 |
| UACH1287 | CH, Chiloe, Puerto Carmen | Locality 39 | -43.14052 | -73.79427 |
| UACH1288 | CH, Chiloe, Puerto Carmen | Locality 39 | -43.14052 | -73.79427 |
| UACH1289 | CH, Chiloe, Puerto Carmen | Locality 39 | -43.14052 | -73.79427 |
| UACH1294 | CH, Chiloe, Puerto Carmen | Locality 39 | -43.14052 | -73.79427 |
| UACH1295 | CH, Chiloe, Puerto Carmen | Locality 39 | -43.14052 | -73.79427 |
| UACH1331 | CH, Chiloe, Puerto Carmen | Locality 39 | -43.14052 | -73.79427 |
| UACH1333 | CH, Chiloe, Puerto Carmen | Locality 39 | -43.14052 | -73.79427 |
| UACH1334 | CH, Chiloe, Puerto Carmen | Locality 39 | -43.14052 | -73.79427 |
| UACH1335 | CH, Chiloe, Puerto Carmen | Locality 39 | -43.14052 | -73.79427 |
| UACH1336 | CH, Chiloe, Puerto Carmen | Locality 39 | -43.14052 | -73.79427 |
| UACH1337 | CH, Chiloe, Puerto Carmen | Locality 39 | -43.14052 | -73.79427 |
| UACH1346 | CH, Chiloe, Puerto Carmen | Locality 39 | -43.14052 | -73.79427 |
| CNP1136 | AR, Estancia Bajada del Guanaco, Chubut | Locality 40 | -44.10087 | -67.99038 |
| CNP4479 | AR, Lago Fontana, 1 km E RP 57 desde cabaña municipal | Locality 42 | -44.86841 | -71.55591 |
| CNP2254 | AR, Puerto Piojo | Locality 41 | -44.88458 | -65.67531 |
| CNP932 | AR, Estancia La Madrugada, Chubut | Locality 43 | -45.37684 | -69.93445 |
| CNP2156 | AR, Pico Salamanca, Chubut | Locality 44 | -45.57937 | -67.34833 |
| CNP2157 | AR, Pico Salamanca, Chubut | Locality 44 | -45.57937 | -67.34833 |
| CNP2163 | AR, Pico Salamanca, Chubut | Locality 44 | -45.57937 | -67.34833 |
| CNP2210 | AR, Pico Salamanca, Chubut | Locality 44 | -45.57937 | -67.34833 |
| CNP2265 | AR, Pico Salamanca, Chubut | Locality 44 | -45.57937 | -67.34833 |
| CNP1149 | AR, Estancia Los Manantiales, Chubut | Locality 45 | -45.69083 | -67.76698 |
| CNP2902 | AR, Río Ecker, 500 Ea. Casa de Piedra | Locality 46 | -47.12472 | -70.86962 |
| CNP4485 | AR, Cajón del río Chico, 3 km al O de río Oro | Locality 47 | -47.43835 | -72.05314 |
| CNP4488 | AR, Cajón del río Chico, 3 km al O de río Oro | Locality 47 | -47.43835 | -72.05314 |
| CNP4505 | AR, Cajón del río Chico, 3 km al O de río Oro | Locality 47 | -47.43835 | -72.05314 |
| CNP2543 | AR, Estancia Cerro del Paso, Santa Cruz | Locality 48 | -47.89314 | -66.42225 |
| CNP2541 | AR, Estancia Cerro Ventana, Santa Cruz | Locality 49 | -48.01573 | -66.00315 |
| CNP2552 | AR, Estancia Cerro Ventana, Santa Cruz | Locality 49 | -48.01573 | -66.00315 |
| CNP2560 | AR, Estancia Cerro Ventana, Santa Cruz | Locality 49 | -48.01573 | -66.00315 |
| CNP2901 | AR, Estancia Cerro Ventana, Santa Cruz | Locality 49 | -48.01573 | -66.00315 |
| CNP2907 | AR, Estancia Cerro Ventana, Santa Cruz | Locality 49 | -48.01573 | -66.00315 |
| CNP4335 | AR, Estancia Las Tunas | Locality 50 | -48.79447 | -71.14051 |
| CNP4559 | AR, Estancia Las Tunas | Locality 50 | -48.79447 | -71.14051 |
| USNM501220 | CH, Magallanes, Antártica CHna, Isla Wellington, Puerto Edén | Locality 51 | -49.11882 | -74.48543 |
| USNM501221 | CH, Magallanes, Antártica CHna, Isla Wellington, Puerto Edén | Locality 51 | -49.11882 | -74.48543 |
| CNP2557 | AR, 4 km W Punta Quilla s/RP 288, Santa Cruz | Locality 52 | -50.11757 | -68.42044 |
| CNP2598 | AR, Cañadón alfa, Ea. Río Cullen | Locality 53 | -52.88278 | -68.47841 |
| UACH2119 | CH, Quinta Emilia, Punta Arenas, Magallanes | Locality 54 | -53.11628 | -70.91358 |
| UACH2120 | CH, Quinta Emilia, Punta Arenas, Magallanes | Locality 54 | -53.11628 | -70.91358 |
| UACH2124 | CH, Quinta Emilia, Punta Arenas, Magallanes | Locality 54 | -53.11628 | -70.91358 |
| UACH2127 | CH, Quinta Emilia, Punta Arenas, Magallanes | Locality 54 | -53.11628 | -70.91358 |
| CNP2535 | AR, Laguna Kosovo, Tierra del Fuego, AR | Locality 55 | -53.81799 | -67.77131 |
| CNP2550 | AR, Estancia San Martín, Río Grande | Locality 56 | -53.83325 | -67.64437 |
| FMNH124544 | CH, Magallanes, Isla Capitán Aracena, Bahía Morris, 50 m | Locality 57 | -54.17352 | -71.33625 |
| MACN28506 | AR, Isla de los Estados, Bahía Francklin | Locality 58 | -54.75832 | -63.97954 |
| CNP2534 | AR, Glaciar Le Martial | Locality 59 | -54.78965 | -68.39495 |
| CNP2551 | AR, Glaciar Le Martial | Locality 59 | -54.78965 | -68.39495 |
| CNP2563 | AR, Glaciar Le Martial | Locality 59 | -54.78965 | -68.39495 |
| CNP2664 | AR, Glaciar Le Martial | Locality 59 | -54.78965 | -68.39495 |
| CNP2565 | AR, Puesto Río Milna | Locality 60 | -54.80506 | -68.49591 |
| CNP2602 | AR, CADIC | Locality 61 | -54.82369 | -68.31719 |
| CNP2608 | AR, CADIC | Locality 61 | -54.82369 | -68.31719 |
| CNP2618 | AR, CADIC | Locality 61 | -54.82369 | -68.31719 |
| CNP2620 | AR, CADIC | Locality 61 | -54.82369 | -68.31719 |
| CNP2654 | AR, CADIC | Locality 61 | -54.82369 | -68.31719 |
| CNP2655 | AR, CADIC | Locality 61 | -54.82369 | -68.31719 |
| CNP2683 | AR, CADIC | Locality 61 | -54.82369 | -68.31719 |
| MACN-Ma27824 | AR, Arroyo Piloto, P.N. Tierra del Fuego | Locality 62 | -54.84383 | -68.55453 |
| MACN-Ma27827 | AR, Arroyo Piloto, P.N. Tierra del Fuego | Locality 62 | -54.84383 | -68.55453 |
| MACN-Ma27841 | AR, Arroyo Piloto, P.N. Tierra del Fuego | Locality 62 | -54.84383 | -68.55453 |
| MACN-Ma27844 | AR, Arroyo Piloto, P.N. Tierra del Fuego | Locality 62 | -54.84383 | -68.55453 |
| MACN-Ma27845 | AR, Arroyo Piloto, P.N. Tierra del Fuego | Locality 62 | -54.84383 | -68.55453 |
| MACN-Ma27848 | AR, Arroyo Piloto, P.N. Tierra del Fuego | Locality 62 | -54.84383 | -68.55453 |
| MACN-Ma27850 | AR, Arroyo Piloto, P.N. Tierra del Fuego | Locality 62 | -54.84383 | -68.55453 |
| MACN-Ma27851 | AR, Arroyo Piloto, P.N. Tierra del Fuego | Locality 62 | -54.84383 | -68.55453 |
| MACN-Ma27853 | AR, Arroyo Piloto, P.N. Tierra del Fuego | Locality 62 | -54.84383 | -68.55453 |
| MACN-Ma27854 | AR, Arroyo Piloto, P.N. Tierra del Fuego | Locality 62 | -54.84383 | -68.55453 |
| MACN-Ma27855 | AR, Arroyo Piloto, P.N. Tierra del Fuego | Locality 62 | -54.84383 | -68.55453 |
| USNM482127 | AR, Isla de los Estados, Bahía Capitán Canepa | Locality 63 | -54.85797 | -64.62608 |
| USNM 482128 | AR, Isla de los Estados, Bahía Capitán Canepa | Locality 63 | -54.85797 | -64.62608 |
| USNM 482129 | AR, Isla de los Estados, Bahía Capitán Canepa | Locality 63 | -54.85797 | -64.62608 |
| USNM 482144 | AR, Isla de los Estados, Bahía Capitán Canepa | Locality 63 | -54.85797 | -64.62608 |
| CNP2596 | CH, Estancia Harberton | Locality 64 | -54.87215 | -67.42443 |
| CNP2632 | CH, Estancia Haberton | Locality 64 | -54.87215 | -67.42443 |
| CNP2671 | CH, Estancia Haberton | Locality 64 | -54.87215 | -67.42443 |
| CNP2932 | CH, Estancia Harberton | Locality 64 | -54.87215 | -67.42443 |

**Table S2.** Results of the Principal Component Analysis (PCA) performed to explore cranial differences among populations of *Abrothrix olivacea* distributed in different environmental units. Contributions of cranial variables to the first three principal components (PC) are shown, together with eigenvalues and percentages of explained variance.

| Cranial measurements | PC 1 |  | PC 2 |  | PC 3 |
| --- | --- | --- | --- | --- | --- |
| SL: skull length | 0.939 |  | -0.251 |  | 0.116 |
| CIL: condylo-incisive length | 0.982 |  | -0.003 |  | -0.018 |
| ZB: zygomatic breadth | 0.849 |  | 0.355 |  | -0.300 |
| BB: braincase breadth | 0.608 |  | 0.349 |  | -0.247 |
| ParL: parietal length | 0.946 |  | -0.035 |  | 0.033 |
| IL: incisive foramina length | 0.815 |  | -0.082 |  | 0.112 |
| IW: incisive foramina width | 0.554 |  | -0.170 |  | 0.093 |
| DL: diastema length | 0.809 |  | -0.305 |  | 0.142 |
| TRL: maxillary toothrow length | 0.577 |  | 0.437 |  | -0.238 |
| PW.M1: palatal width at M1 | 0.814 |  | 0.256 |  | -0.209 |
| PW.M3: palatal width at M3 | 0.616 |  | 0.268 |  | 0.050 |
| ZPW: zygomatic plate width | 0.702 |  | 0.306 |  | -0.136 |
| NL: nasal length | 0.642 |  | -0.451 |  | -0.276 |
| NW: nasal width | 0.380 |  | 0.004 |  | 0.100 |
| RW: rostrum width | 0.601 |  | 0.360 |  | -0.456 |
| FSW: frontal sinus width | 0.513 |  | 0.270 |  | -0.038 |
| IB: interorbital breadth | 0.244 |  | -0.218 |  | 0.369 |
| FL: frontal length | 0.519 |  | 0.528 |  | 0.524 |
| PalL: palatilar length | 0.566 |  | 0.166 |  | 0.211 |
| WMF: mesopterygoid fossa width | 0.217 |  | -0.290 |  | 0.284 |
| Eigenvalues | 3.706 |  | 0.407 |  | 0.257 |
| Percentage of explained variance (EV) | 67.550 |  | 7.423 |  | 4.690 |

**Table S3.** Results of the second Discriminant Function Analysis (DFA) performed to explore cranial differences among populations of *Abrothrix olivacea* and differences of the samples from the geographic distributions of the nominal forms *hershkovitzi* (Capitan Aracena Island), *llanoi* (de los Estados Island), and *markhami* (Wellington Island). Loadings of the cranial variables to the first three discriminant functions (DF), F values, and significance (*p* value) obtained for each variable are shown. Percentage of variance explained by each DF and their eigenvalues are provided at the end of the table.

| Cranial measurements | Linear discriminant functions | | | F value |  | *p* value |
| --- | --- | --- | --- | --- | --- | --- |
|  | DF1 | DF2 | DF3 |  |  |  |
| SL: skull length | 36.787 | -14.365 | 2.474 | 12.796 |  | 2.20E-16 |
| CIL: condylo-incisive length | -28.796 | 20.414 | -31.522 | 22.095 |  | 2.20E-16 |
| ZB: zygomatic breadth | 8.202 | 5.085 | 24.060 | 22.589 |  | 2.20E-16 |
| BB: braincase breadth | -4.790 | 6.445 | -10.368 | 12.089 |  | 1.57E-15 |
| ParL: parietal length | -14.437 | -6.288 | 1.705 | 26.067 |  | 2.20E-16 |
| IL: incisive foramina length | -3.990 | -5.857 | 6.360 | 21.067 |  | 2.20E-16 |
| IW: incisive foramina width | -0.504 | -3.236 | 2.540 | 9.1447 |  | 8.86E-12 |
| DL: diastema length | 8.897 | 1.498 | -7.982 | 11.07 |  | 2.94E-14 |
| TRL: maxillary toothrow length | -6.336 | 3.301 | -4.300 | 25.872 |  | 2.20E-16 |
| PW.M1: palatal width at M1 | -8.144 | -6.900 | -9.700 | 26.681 |  | 2.20E-16 |
| PW.M3: palatal width at M3 | 0.327 | 5.938 | 0.196 | 12.887 |  | 2.20E-16 |
| ZPW: zygomatic plate width | -1.902 | 2.371 | 0.872 | 18.875 |  | 2.20E-16 |
| NL: nasal length | 2.099 | -5.648 | 10.017 | 5.8642 |  | 2.41E-07 |
| NW: nasal width | 4.807 | 1.197 | 1.865 | 4.2218 |  | 4.58E-05 |
| RW: rostrum width | -2.609 | 3.919 | 0.718 | 16.928 |  | 2.20E-16 |
| FSW: frontal sinus width | -0.255 | -2.024 | 2.568 | 9.7106 |  | 1.62E-12 |
| IB: interorbital breadth | 2.513 | -10.179 | -4.957 | 13.177 |  | 2.20E-16 |
| FL: frontal length | -3.562 | -8.103 | 4.355 | 11.511 |  | 8.21E-15 |
| PalL: palatilar length | -6.584 | -3.525 | 4.068 | 21.375 |  | 2.20E-16 |
| WMF: mesopterygoid fossa width | -1.876 | -3.235 | -0.216 | 13.155 |  | 2.20E-16 |
| Percentage of explained variance (EV) | 52.24 | 24.14 | 9.16 |  |  |  |
| Eigenvalues | 10.055 | 6.836 | 4.210 |  |  |  |

**Figure S1.** Orthogonal projections of the three first Principal Components (PC), which cumulate 79.66% of the explained variance contained by the cranial variables considered (see Table S1). Specimens were grouped according to environmental units present in their sampling locality; in turns, groups are colored as is depicted in the legend. Abbreviation of the environmental units and their corresponding *n* are as follow: CS, Chilean shrubland = 23; DF, dry forest = 45; HF, humid forest = 81; LM, Low monte = 1; PP, Andean steppe or Prepuna = 13; PS, Patagonian steppe 30; TF, temperate forest = 42. In the right panel are biplots of this PCA analysis depicting the association of cranial variables to the principal components considered.


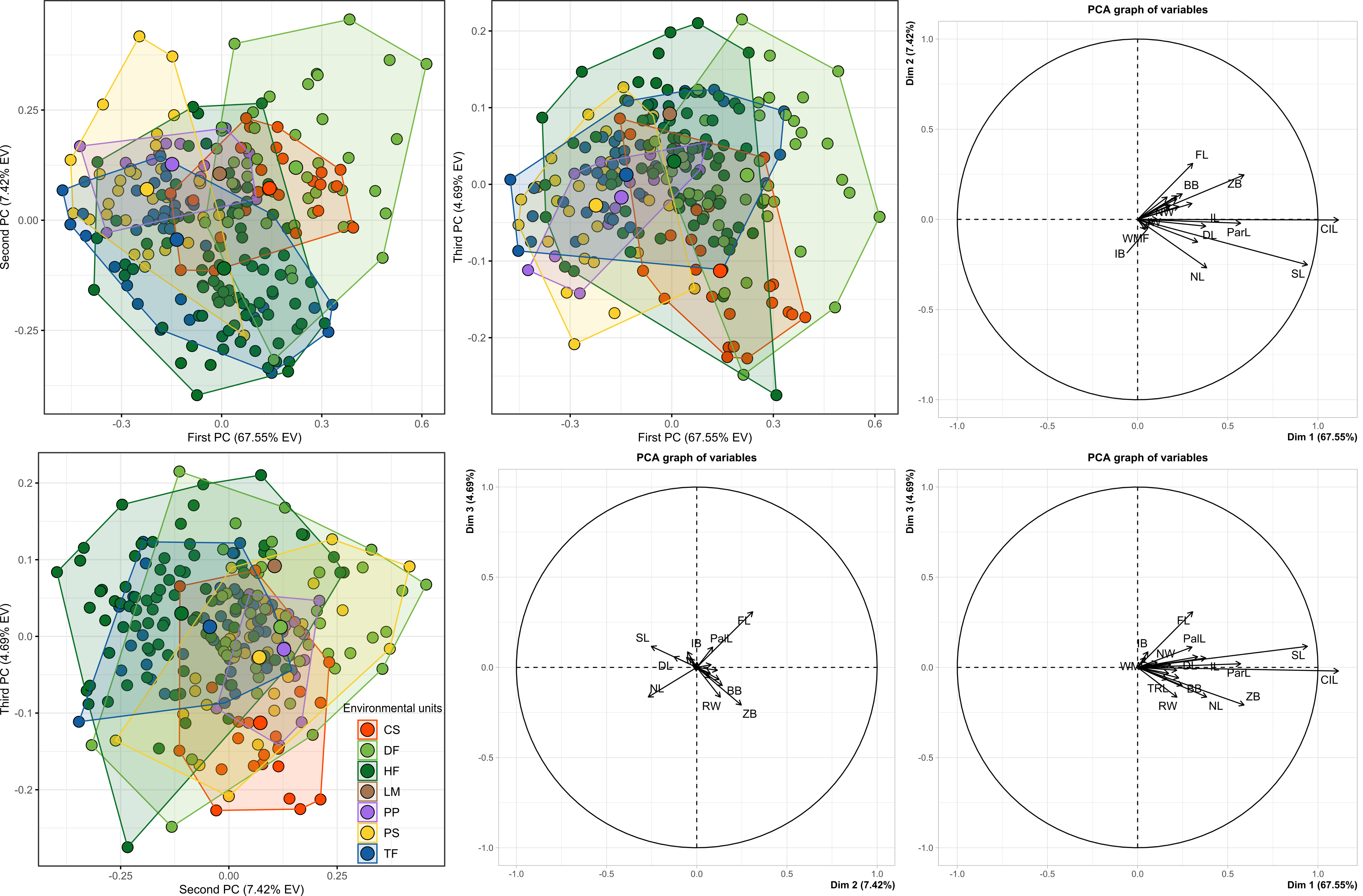


**Figure S2.** Orthogonal projection of the three first Discriminant Functions (DF), which explain 85.54% of the variance accounted by the DFA, based on the cranial variables considered (see Table S2). Specimens were grouped according to the environmental units to which correspond their sampling locality, and the insular localities from which the nominal forms *hershkovitzi*, *llanoi*, and *markhami* come from (see above). Groups are colored as is depicted in the legend (see also Figure 1). Abbreviations of the environmental units are as follow: CS, Chilean shrubland; DF, dry forest; HF, humid forest; LM, Low monte; PP, Andean steppe or Prepuna; PS, Patagonian steppe; TF, temperate forest. As is depicted in the Figure 1, evaluated insular localities occurred along Temperate forests. Percentages of correct a priori assignation of specimens to each group are as follow: CS = 86.96%; DF = 95.55%; HF = 90.12%; LM = 100%; PP = 92.31%; PS = 83.33%; TF = 73.53%; TF_C. Aracena = 100%; TF_I. Estados = 100%; TF_I. Wellington = 100%.


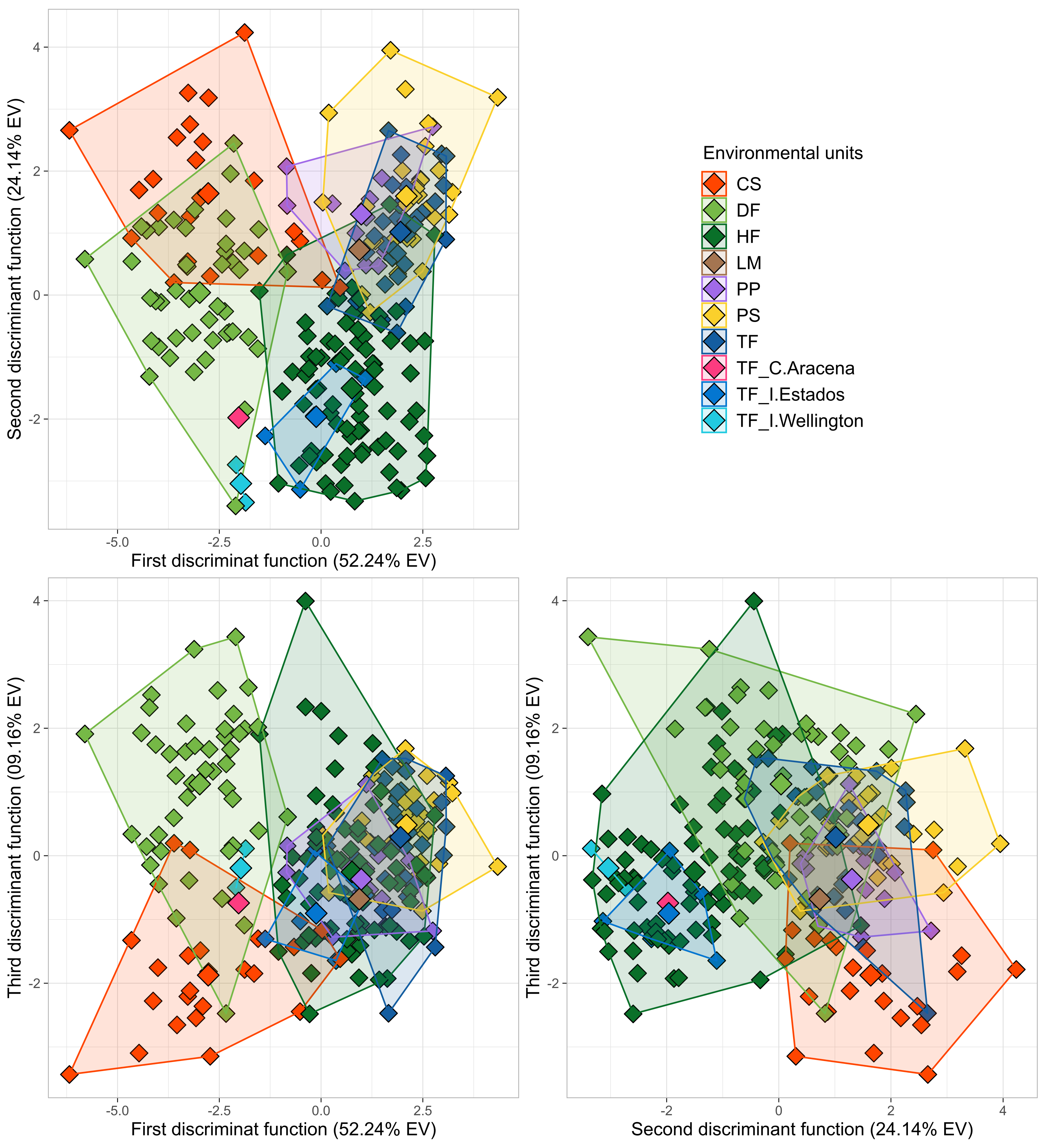


**Table S3.** Results of linear regression analyses conducted showing associations the scores of cranial variables on PC 1 (summary of cranial size) and PC 2 (summary of cranial shape) alongside to the uncorrelated environmental variables and geospatial variables considered. Patterns of correlation are depicted for the entire dataset of cranial measurements (i.e., at species level) and for those resulting after dividing the morphological dataset according to the environmental units in which the species inhabit (i.e., at population). Coefficients of correlation (*r*) and their respective *p*-value is indicated by each associations analyzed. Abbreviations of the environmental units are as follow: CS, Chilean shrubland; DF, dry forest; HF, humid forest; LM, Low monte; PP, Andean steppe or Prepuna; PS, Patagonian steppe; TF, temperate forest. As is depicted in the Figure 1, evaluated insular localities occurred along Temperate forests.

|  | At species level |  | At environmental units | | | | | |
| --- | --- | --- | --- | --- | --- | --- | --- | --- |
|  | *Abrothrix olivacea* |  | CS | DF | HF | PP | PS | TF |
| *PC 1 vs. Environmental and Geospatial variables* | | | | | | | | |
| Bio 01: Annual Mean Temperature | *R* = 0.51; *p* = 0.00 |  | *R* = 0.26; *p* = 0.23 | *R* = 0.13; *p* = 0.41 | *R* = 0.52; *p* = 0.00 | *R* = -0.05; *p* = 0.87 | *R* = -0.26, *p* = 0.16 | *R* = -0.37; *p* = 0.02 |
| Bio 03: Isothermality (BIO2/BIO7) (×100) | *R* = 0.35; *p* = 0.00 |  | *R* = 0.15; *p* = 0.49 | *R* = -0.03; *p* = 0.84 | *R* = 0.23; *p* = 0.07 | *R* = =0.02; *p* = 0.95 | *R* = 0.23; *p* = 0.21 | *R* = 0.13; *p* = 0.44 |
| Bio 08: Mean Temperature of Wettest Quarter | *R* = 0.47; *p* = 0.00 |  | *R* = 0.28; *p* = 0.21 | *R* = 0.03; *p* = 0.84 | *R* = 0.41; *p* = 0.00 | *R* = -0.04; *p* = 0.89 | *R* = 0.04; *p* = 0.84 | *R* = 0.11; *p* = 0.52 |
| Bio 12: Annual Precipitation | *R* = 0.21; *p* = 0.00 |  | *R* = 0.41; *p* = 0.06 | *R* = 0.07; *p* = 0.63 | *R* = 0.41; *p* = 0.00 | *R* = 0.55; *p* = 0.05 | *R* = 0.38; *p* = 0.04 | *R* = 0.59; *p* = 0.00 |
| Bio 15: Precipitation Seasonality (Coefficient of Variation) | *R* = 0.61; *p* = 0.00 |  | *R* = 0.18; *p* = 0.41 | *R* = 0.14; *p* = 0.52 | *R* = 0.35; *p* = 0.01 | *R* = 0.59; *p* = 0.03 | *R* = -0.10; *p* = 0.59 | *R* = 0.21; *p* = 0.19 |
| Bio 16: Precipitation of Wettest Quarter | *R* = 0.33; *p* = 0.00 |  | *R* = 0.42; *p* = 0.06 | *R* = 0.12; *p* = 0.51 | *R* = 0.49; *p* = 0.00 | *R* = 0.58; *p* = 0.04 | *R* = 0.27; *p* = 0.15 | *R* = 0.61; *p* = 0.00 |
| Humidity | *R* = 0.06; *p* = 0.33 |  | *R* = 0.39; *p* = 0.05 | *R* = 0.03; *p* = 0.82 | *R* = 0.22; *p* = 0.04 | *R* = 0.04; *p* = 0.89 | *R* = 0.43; *p* = 0.02 | *R* = 0.66; *p* = 0.00 |
| Evapotranspiration | *R* = 0.09; *p* = 0.14 |  | *R* = -0.44; *p* = 0.35 | *R* = -0.01; *p* = 0.94 | *R* = -0.07; *p* = 0.53 | *R* = 0.01; *p* = 0.98 | *R* = -0.07; *p* = 0.72 | *R* = -0.31; *p* = 0.06 |
| Primary productivity | *R* = 0.38; *p* = 0.00 |  | *R* = 0.38; *p* = 0.07 | *R* = 0.06; *p* = 0.67 | *R* = 0.11; *p* = 0.33 | *R* = -0.53; *p* = 0.06 | *R* = 0.24; *p* = 0.21 | *R* = 0.06; *p* = 0.71 |
| Elevation | *R* = -0.10; *p* = 0.13 |  | *R* = -0.33; *p* = 0.13 | *R* = -0.11; *p* = 0.48 | *R* = -0.38; *p* = 0.00 | *R* = 0.03; *p* = 0.92 | *R* = 0.28; *p* = 0.13 | *R* = 0.53; *p* = 0.00 |
| Latitude | *R* = 0.51; *p* = 0.00 |  | *R* = -0.44; *p* = 0.04 | *R* = 0.04; *p* = 0.78 | *R* = 0.44; *p* = 0.00 | *R* = -0.21; *p* = 0.48 | *R* = 0.05; *p* = 0.81 | *R* = 0.44; *p* = 0.00 |
| Longitude | *R* = -0.41; *p* = 0.00 |  | *R* = -0.43; *p* = 0.04 | *R* = 0.24; *p* = 0.12 | *R* = -0.24; *p* = 0.07 | *R* = -0.28; *p* = 0.35 | *R* = 0.17; *p* = 0.36 | *R* = -0.10; *p* = 0.58 |
| *PC 2 vs. Environmental and Geospatial variables* | | | | | | | | |
| Bio 01: Annual Mean Temperature | *R* = 0.17; *p* = 0.00 |  | *R* = 0.09; *p* = 0.66 | *R* = 0.11; *p* = 0.46 | *R* = -0.19; *p* = 0.08 | *R* = -0.15; *p* = 0.62 | *R* = 0.34; *p* = 0.07 | *R* = 0.06; *p* = 0.71 |
| Bio 03: Isothermality (BIO2/BIO7) (×100) | *R* = 0.34; *p* = 0.00 |  | *R* = 0.15; *p* = 0.48 | *R* = -0.22; *p* = 0.14 | *R* = 0.45; *p* = 0.00 | *R* = 0.07; *p* = 0.82 | *R* = -0.45; *p* = 0.01 | *R* = -0.12; *p* = 0.46 |
| Bio 08: Mean Temperature of Wettest Quarter | *R* = 0.00; *p* = 0.92 |  | *R* = 0.11; *p* = 0.63 | *R* = 0.08; *p* = 0.59 | *R* = -0.36; *p* = 0.00 | *R* = -0.15; *p* = 0.63 | *R* = 0.48; *p* = 0.00 | *R* = -0.32; *p* = 0.05 |
| Bio 12: Annual Precipitation | *R* = -0.47; *p* = 0.00 |  | *R* = 0.25; *p* = 0.25 | *R* = 0.21; *p* = 0.19 | *R* = -0.08; *p* = 0.46 | *R* = -0.3; *p* = 0.33 | *R* = -0.33; *p* = 0.07 | *R* = -0.39; *p* = 0.01 |
| Bio 15: Precipitation Seasonality (Coefficient of Variation) | *R* = 0.31; *p* = 0.00 |  | *R* = 0.04; *p* = 0.85 | *R* = -0.06; *p* = 0.67 | *R* = 0.13; *p* = 0.23 | *R* = -0.24; *p* = 0.44 | *R* = -0.08; *p* = 0.65 | *R* = -0.45; *p* = 0.04 |
| Bio 16: Precipitation of Wettest Quarter | *R* = -0.37; *p* = 0.00 |  | *R* = 0.25; *p* = 0.25 | *R* = 0.25; *p* = 0.10 | *R* = 0.00; *p* = 0.99 | *R* = -0.29; *p* = 0.34 | *R* = -0.37; *p* = 0.04 | *R* = -0.42; *p* = 0.00 |
| Humidity | *R* = -0.42; *p* = 0.00 |  | *R* = 0.25; *p* = 0.25 | *R* = 0.14; *p* = 0.35 | *R* = -0.01; *p* = 0.90 | *R* = 0.16; *p* = 0.62 | *R* = -0.32; *p* = 0.09 | *R* = -0.34; *p* = 0.03 |
| Evapotranspiration | *R* = 0.51; *p* = 0.00 |  | *R* = -0.25; *p* = 0.26 | *R* = -0.25; *p* = 0.10 | *R* = 0.51; *p* = 0.00 | *R* = -0.12; *p* = 0.69 | *R* = 0.22; *p* = 0.29 | *R* = 0.05; *p* = 0.74 |
| Primary productivity | *R* = -0.16; *p* = 0.02 |  | *R* = 0.24; *p* = 0.27 | *R* = 0.25; *p* = 0.09 | *R* = 0.24; *p* = 0.03 | *R* = -0.06; *p* = 0.82 | *R* = 0.38; *p* = 0.039 | *R* = -0.36; *p* = 0.02 |
| Elevation | *R* = 0.22; *p* = 0.00 |  | *R* = -0.14; *p* = 0.51 | *R* = -0.24; *p* = 0.11 | *R* = 0.40; *p* = 0.00 | *R* = 0.17; *p* = 0.57 | *R* = -0.37; *p* = 0.04 | *R* = -0.08; *p* = 0.59 |
| Latitude | *R* = 0.26; *p* = 0.00 |  | *R* = -0.27; *p* = 0.21 | *R* = -0.17; *p* = 0.27 | *R* = 0.16; *p* = 0.14 | *R* = 0.39; *p* = 0.10 | *R* = -0.22; *p* = 0.24 | *R* = -0.26; *p* = 0.11 |
| Longitude | *R* = 0.25; *p* = 0.00 |  | *R* = -0.22; *p* = 0.31 | *R* = -0.049; *p* = 0.75 | *R* = 0.53; *p* = 0.00 | *R* = 0.41; *p* = 0.16 | *R* = 0.21; *p* = 0.28 | *R* = -0.14; *p* = 0.38 |

**Figure S3.** Associations of the first Principal Component with environmental and geospatial variables. Linear regression analysis shows the association scores of cranial variables on PC 1 (summary of cranial size) alongside to the uncorrelated environmental variables and geospatial variables considered. Correlation coefficients (*R*) and associated *p*-values are shown for each analysis.


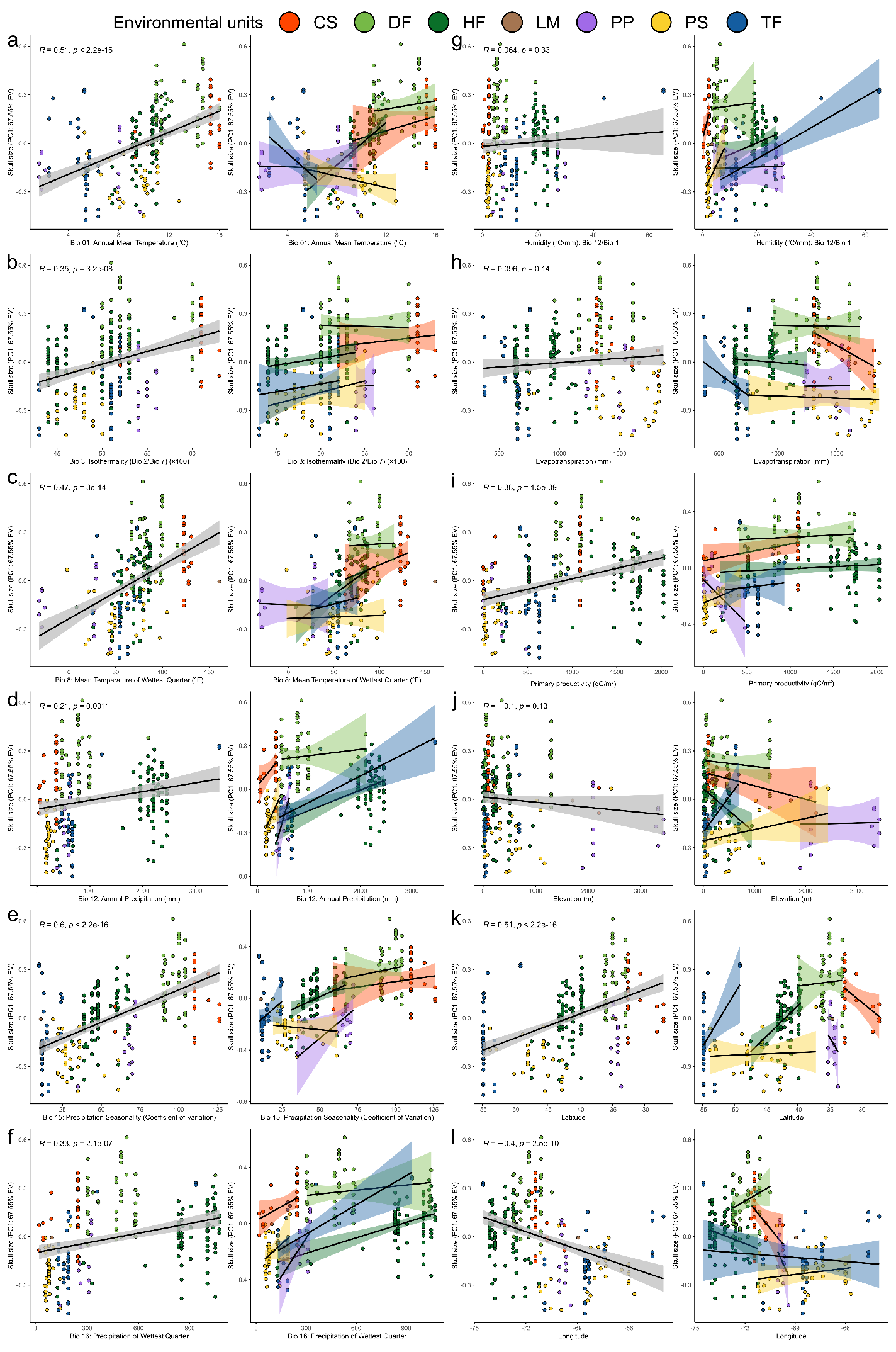


**Figure S4.** Associations of the second principal component with environmental and geospatial variables. Linear regression analysis shows association scores of cranial variables on PC 2 (summary of cranial shape) alongside to the uncorrelated environmental variables and geospatial variables considered. Correlation coefficients (*R*) and associated *p*-values are shown for each analysis.


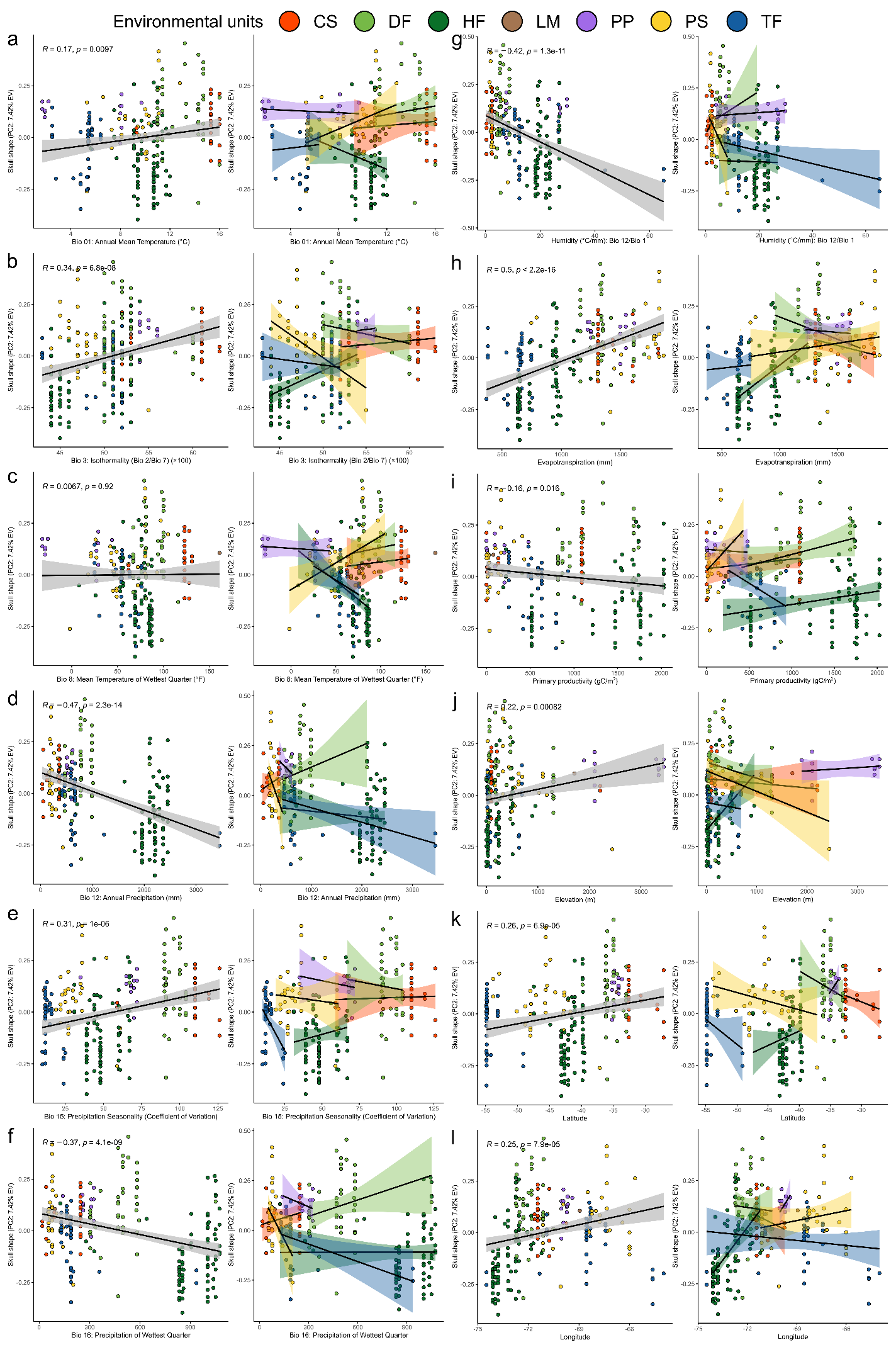


**Figure S5**. Results of Mantel tests performed to explore associations between genetic (proxy of neutral differentiation) and Euclidean morphological distances calculate between pairs of a reduce set of 29 localities with mitochondrial and morphological data available. The results are based on Tajima-Nei distance and Tamura-Nei distance. Spearman correlation coefficients (R) and associated *p*-values are shown for each Mantel test implementation. According to Bacigalupe et al. (2008), a pattern of positive correlation between morphological and neutral genetic distances indicates that genetic drift is likely responsible for the morphological differentiation of populations. In this case, there is not a positive correlation between the levels of neutral genetic and morphological differentiation among populations. Therefore, the role of genetic drift in the process of morphological differentiation of the populations considered can be ruled out. Given the limited geographic coverage that founded these analyses, the pattern evidenced by these results cannot be generalized to the entire geographic distribution of *Abrothrix olivacea*. Moreover, its generalization to the entire Patagonian region could be premature, due to the representative limitations associated to the dataset used (Bacigalupe, personal communication).


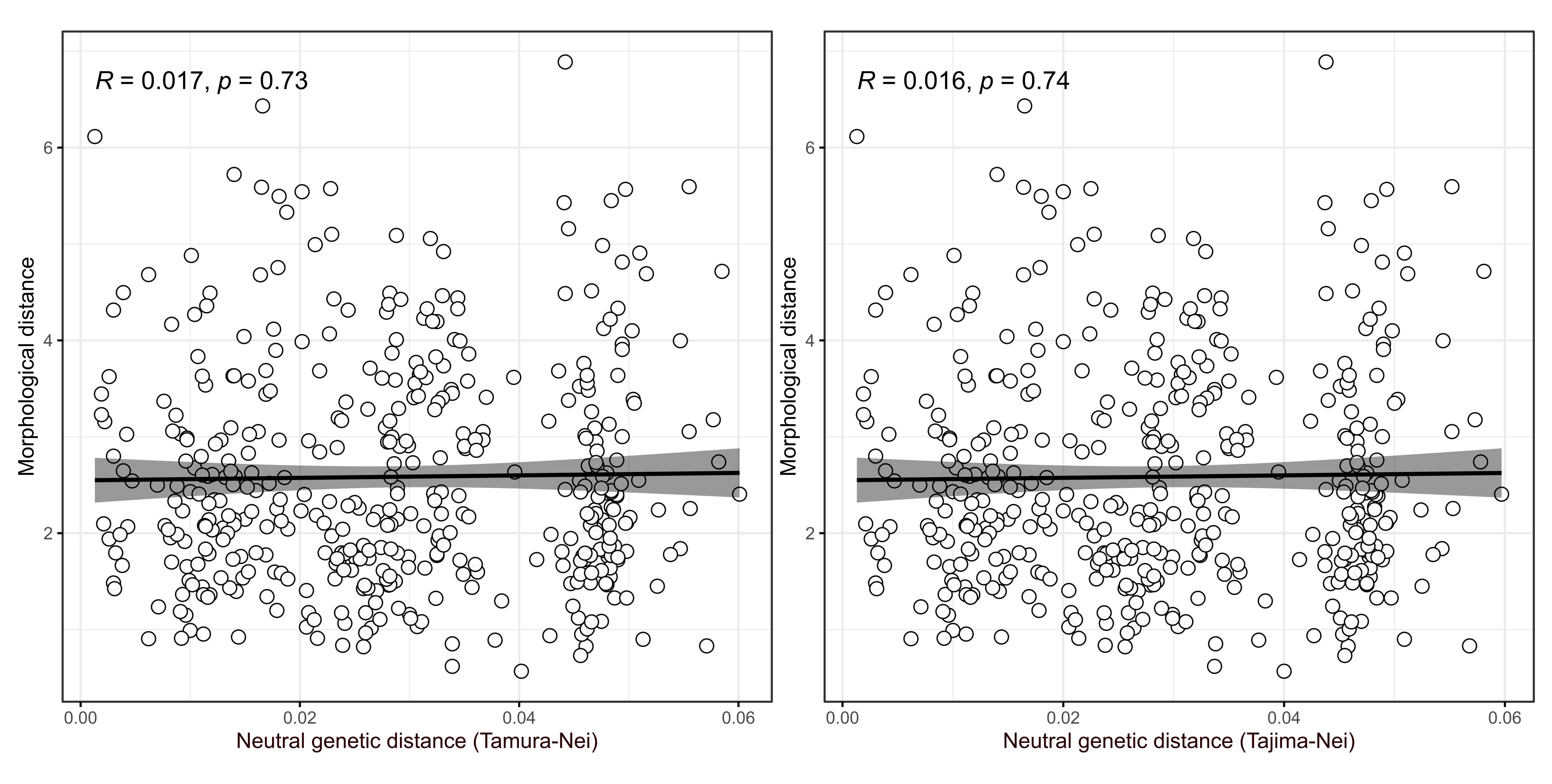


**Figure S6.** Cranial size variation within and among populations of *Abrothrix olivacea*. Plots show individual scores for the first principal component (upper panel) and size index estimated from the geometric means of the cranial measurements considered (lower panel). Population samples are colored according to environmental units (see Figure 1: CS, Chilean shrubland; DF, dry forest; HF, humid forest; LM, Low monte; PP, Andean steppe/Prepuna; PS, Patagonian steppe; TF, temperate forest); samples from insular regions are individualized (e.g., Capitán Aracena, C.Aracena; Chiloé, Chiloe.I; los Estados, I.Estados; Tierra del Fuego, T.Fuego; Wellington, I.Wellington). Codification of each population is in the legend.


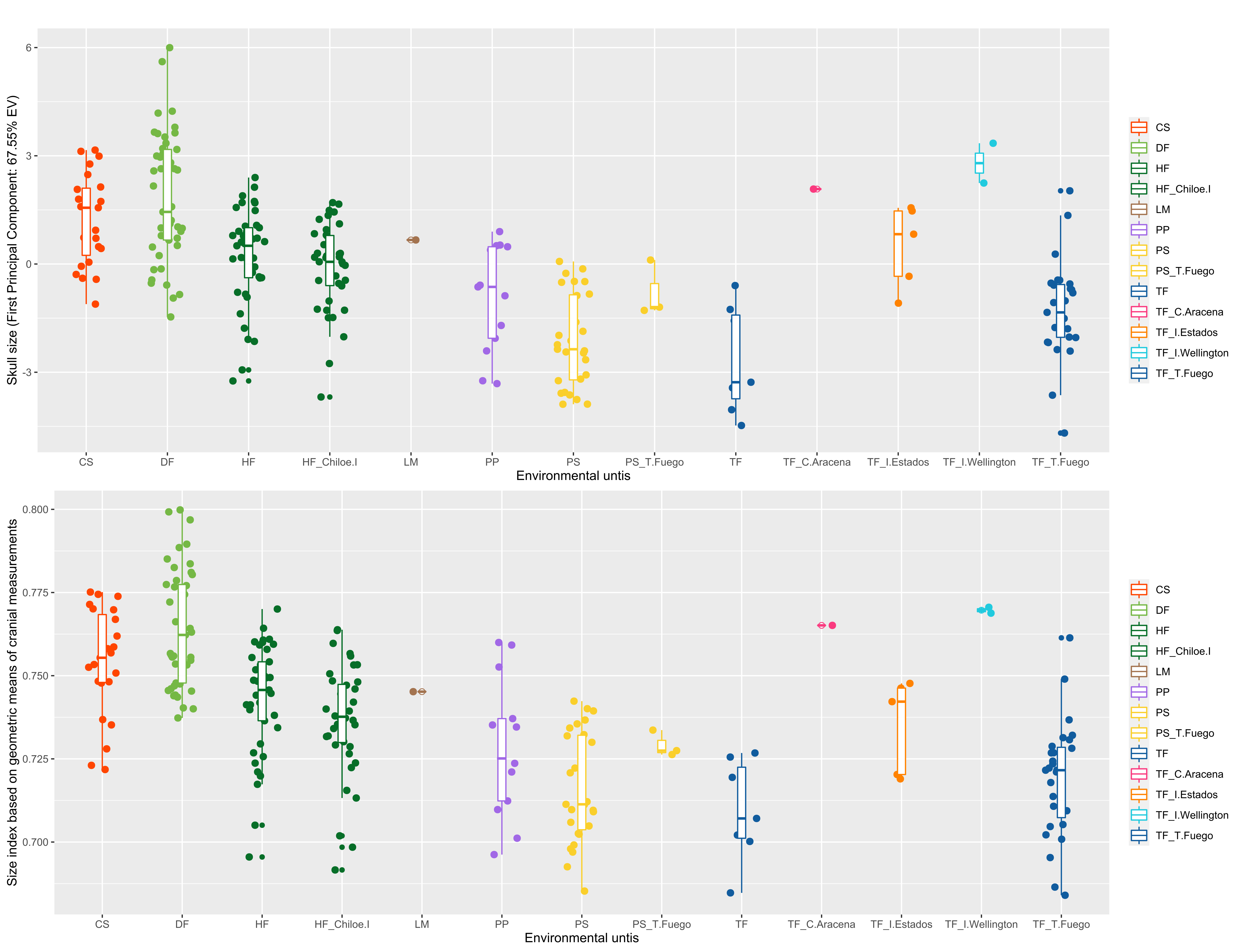

Supplement: Supplemental Information 2 [file peerj-11-15200-s002.docx]
